# Biomarkers and experimental models for cancer immunology investigation

**DOI:** 10.1002/mco2.437

**Published:** 2023-12-02

**Authors:** Hengyi Xu, Ziqi Jia, Fengshuo Liu, Jiayi Li, Yansong Huang, Yiwen Jiang, Pengming Pu, Tongxuan Shang, Pengrui Tang, Yongxin Zhou, Yufan Yang, Jianzhong Su, Jiaqi Liu

**Affiliations:** ^1^ State Key Laboratory of Molecular Oncology National Cancer Center /National Clinical Research Center for Cancer/Cancer Hospital Chinese Academy of Medical Sciences and Peking Union Medical College Beijing China; ^2^ Eight‐year MD Program School of Clinical Medicine, Chinese Academy of Medical Sciences and Peking Union Medical College Beijing China; ^3^ Department of Breast Surgical Oncology National Cancer Center/National Clinical Research Center for Cancer/Cancer Hospital Chinese Academy of Medical Sciences and Peking Union Medical College Beijing China; ^4^ School of Medicine Tsinghua University Beijing China; ^5^ Oujiang Laboratory Zhejiang Lab for Regenerative Medicine, Vision, and Brain Health Wenzhou Zhejiang China

**Keywords:** biomarker, cancer immunotherapy, experimental model, lab‐on‐a‐chip devices, three‐dimensional model

## Abstract

The rapid advancement of tumor immunotherapies poses challenges for the tools used in cancer immunology research, highlighting the need for highly effective biomarkers and reproducible experimental models. Current immunotherapy biomarkers encompass surface protein markers such as PD‐L1, genetic features such as microsatellite instability, tumor‐infiltrating lymphocytes, and biomarkers in liquid biopsy such as circulating tumor DNAs. Experimental models, ranging from 3D in vitro cultures (spheroids, submerged models, air–liquid interface models, organ‐on‐a‐chips) to advanced 3D bioprinting techniques, have emerged as valuable platforms for cancer immunology investigations and immunotherapy biomarker research. By preserving native immune components or coculturing with exogenous immune cells, these models replicate the tumor microenvironment in vitro. Animal models like syngeneic models, genetically engineered models, and patient‐derived xenografts provide opportunities to study in vivo tumor‐immune interactions. Humanized animal models further enable the simulation of the human‐specific tumor microenvironment. Here, we provide a comprehensive overview of the advantages, limitations, and prospects of different biomarkers and experimental models, specifically focusing on the role of biomarkers in predicting immunotherapy outcomes and the ability of experimental models to replicate the tumor microenvironment. By integrating cutting‐edge biomarkers and experimental models, this review serves as a valuable resource for accessing the forefront of cancer immunology investigation.

## Graphical abstract

The rapid progress in tumor immunotherapies poses challenges for cancer immunology research tools, particularly in terms of the need for high‐efficacy biomarkers and the limited reproducibility of experimental models. Biomarkers currently used in research can be categorized into surface protein markers, genetic features, tumor‐infiltrating lymphocytes, and liquid biopsy biomarkers. Experimental models, such as 3D in vitro cultures and animal models, have emerged as valuable platforms for biomarker research and cancer immunology investigations. 3D in vitro cultures, including spheroid models, submerged models, air–liquid interface models, and organ‐on‐a‐chip systems, offer the ability to replicate tumor‐immune interactions by preserving native immune components or through coculturing with immune cells. Animal models, including syngeneic models, genetically engineered models, and patient‐derived xenografts, offer opportunities to study tumor‐immune interactions in an in vivo setting. Furthermore, humanized animal models provide a means to simulate the human‐specific tumor microenvironment, enabling a more accurate representation of the complex immune responses observed in patients. In summary, the development of high‐efficacy biomarkers and the utilization of advanced experimental models, such as 3D in vitro cultures and animal models, offer promising avenues for studying tumor immunology and advancing cancer immunotherapies.

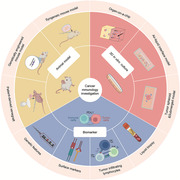

## 1 INTRODUCTION

Significant progress in cancer immunotherapy, including immune checkpoint blockers (ICBs),^1^ tumor vaccines,^2^ and adoptive cell therapies (ACT),^3^ has led to an approximate 10% increase in the 5‐year overall survival (OS) rate across diverse cancer types, such as lymphoma, melanoma, and non‐small cell lung cancer (NSCLC).^4^, ^5^, ^6^ Immunotherapy aims to manipulate the immune system to recognize and eliminate cancer cells.^1^ Rapid development of tumor immunotherapies further pose challenges for the tools utilized in cancer immunology investigations, particularly concerning the availability of highly effective biomarkers.^2^ Currently, guidelines recommend predictive markers are PD‐L1 expression in tumor cells and immune cells^7^ and microsatellite instability (MSI).^8^ However, challenges arise due to insufficient evidence for response prediction based on PD‐L1 expression in early‐stage tumors^9^ and the limited application of MSI.^10^ Other potential prediction biomarkers including tumor‐infiltrating lymphocytes (TILs)^11^ and tumor mutation burden (TMB)^12^ are constantly emerging. The emergence of experimental models that faithfully replicate the in vivo antitumor immune response has become crucial in biomarker research and cancer immunology investigations.^13^, ^14^

For the discovery of reliable immunotherapy biomarkers, 3D in vitro models that can reproduce the tumor microenvironment (TME) and tumor‐immune interactions are necessary.^15^ TME, consisting of immune cells, stroma cells, blood, and lymphatic vessels embedded in a noncellular extracellular matrix (ECM), plays a crucial role in tumor progression, therapeutic response, and patient outcomes.^16^ Though current tumor models such as two‐dimensional (2D) culture,^17^ three‐dimensional (3D) culture,^18^ and patient‐derived xenograft (PDX)^19^ display high reproducibility in terms of tumor cell properties, they have limited capacity to mimic personalized TME. To simulate in vivo tumor‐immune interactions, 2D cultures can be cocultured with various exogenously added heterogeneous cells.^20^, ^21^, ^22^ However, these reconstituted cells often do not originate from the native tumor, and the flat monolayer configuration in 2D cultures fails to replicate the complex 3D morphological structures. Compared with 3D cultures, the biology of oncogenes and tumor suppressors in 2D cultures may be less faithful to their in vivo counterparts.^21^, ^23^

**FIGURE 1:**
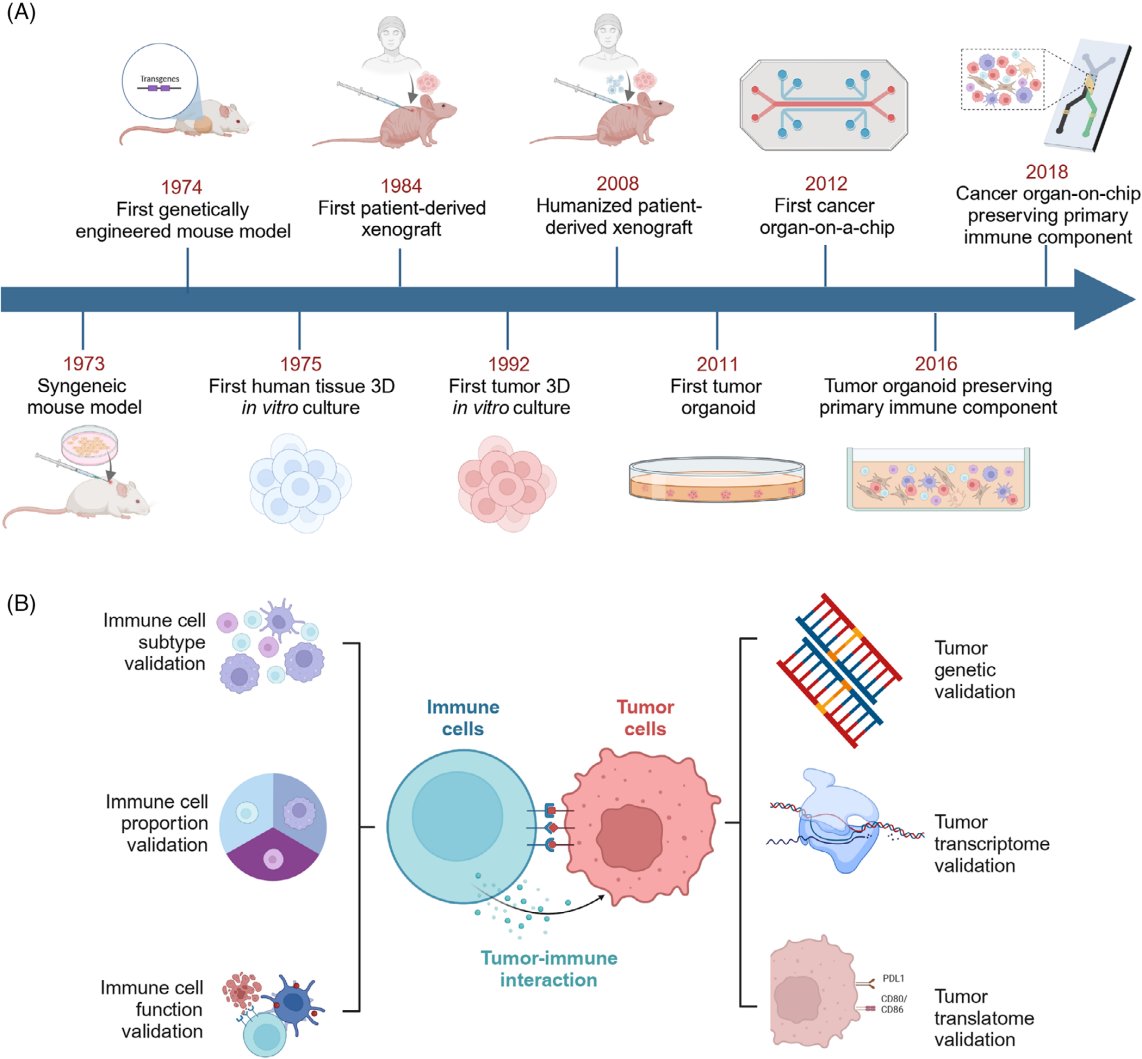
Development and validation: Experimental models for cancer immunology investigations. (A) Early in the 1970s, in vitro models such as spheroid and animal models such as syngeneic models have been utilized in cancer immunology investigations. In the recent decade, experimental models preserving original tumor components emerged, facilitating the research on tumor microenvironment and tumor immunotherapy. (B) Validation of a 3D in vitro tumor‐immune coculture system can be completed through two aspects: tumor components and immune components. Validations of tumor components include three different levels: genetic (DNA mutation), transcriptomic (RNA profiling), and translatomic (surface protein expression). Validations on immune components are composed of immune cell subtype, immune cell proportion, and immune cell function. Figure was created with BioRender.com.

Since the first in vitro 3D culture of human normal tissue was developed in 1975,^24^ the 3D tumor culture has achieved significant development (Figure 1A). Spheroids, defined by tumor multicellular spherical colonies suspended in three dimensions, were described in 1992 on breast cancer, creating the first 3D in vitro tumor model.^25^ In 2011, the first cancer organoid, characterized by 3D tumor cultures retaining histological and genetic features of the primary tumor, was established on colorectal cancer (CRC).^26^ The first cancer‐on‐a‐chip, involving 3D tumor models based on microfluidic systems, was successfully established in 2012.^27^ Over the past decade, there has been a growing interest in in vitro 3D tumor‐immune coculture systems.^28^ Noteworthy, verifications of tumor‐immune interactions encompass both aspects of tumor cells and immune cells (Figure 1B). Additionally, animal models including humanized mouse models provide a valuable platform for evaluating immunotherapies and investigating in vivo TME.^29^ Early in the 1970s, syngeneic mouse model, which involves injecting murine‐derived tumor cell lines into immunocompetent mice, has been constructed for melanoma.^30^ Genetically engineered mouse models (GEMMs), introduced in 1974, enable spontaneous tumor formation in genetically engineered mice.^31^ In 1984, PDXs emerged as the first animal models that directly preserve patient‐derived tumor cells.^32^ In the 21st century, humanized mouse models allow the reconstruction of the human immune system in immunodeficient mice.^33^

Biomarkers and experimental models are interconnected and complementary in cancer immunology investigation.^13^ In this review, we first provide a comprehensive summary of a range of classical and emerging biomarkers. We further clarify their relationship with the underlying tumor immunology mechanisms and various immunotherapies. Subsequently, we classify experimental models into two categories: in vivo and in vitro, and discuss the architecture, features, and applications of each model in the context of tumor immunology and immunotherapy. Special attention is given to exploring different approaches for TME reconstruction. Furthermore, we summarize the role of biomarkers and experimental models in cancer immunology investigation and outline future directions.

## 2 BIOMARKERS FOR TUMOR IMMUNOLOGY INVESTIGATION

**FIGURE 2:**
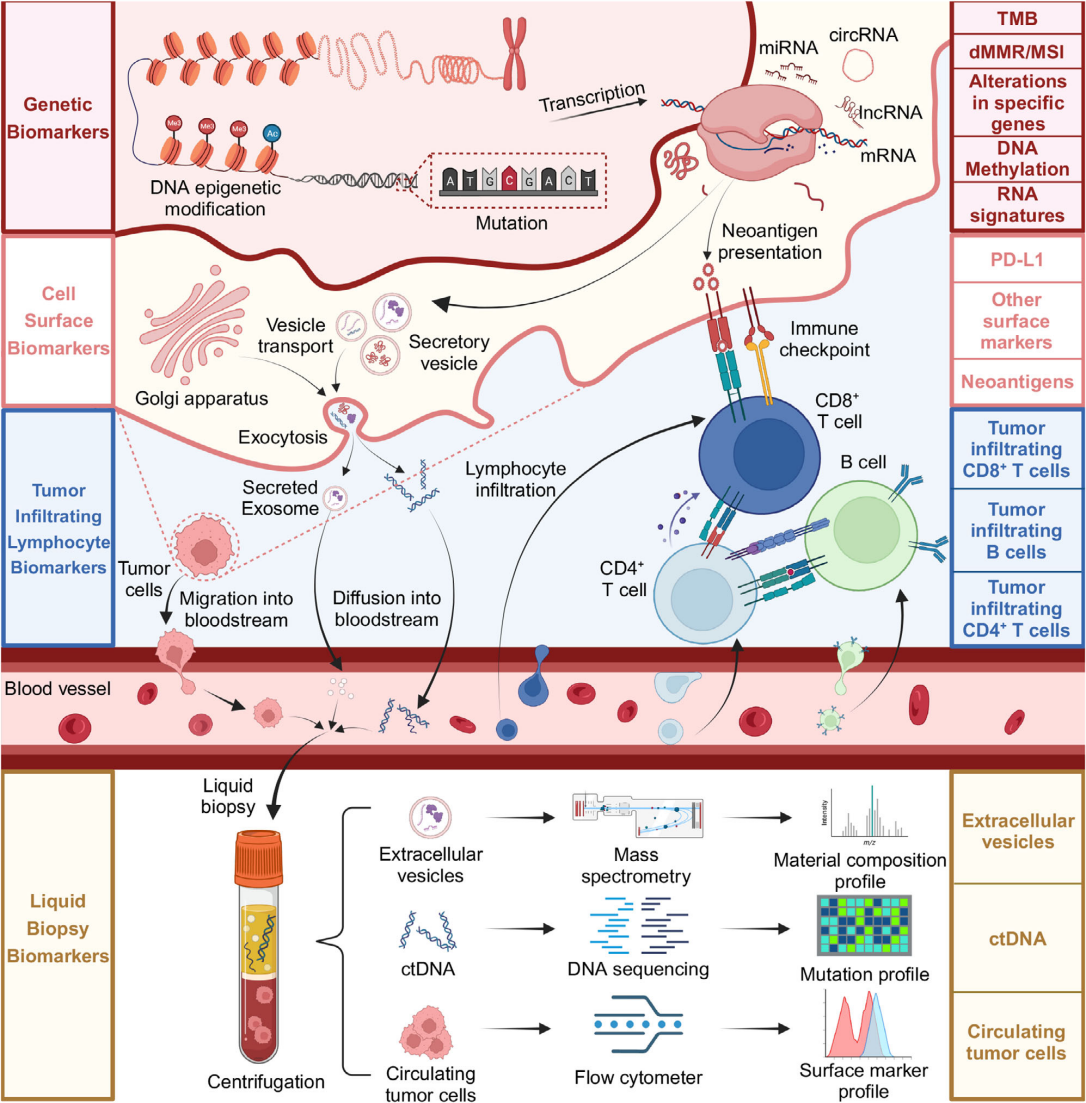
Biomarkers used in cancer immunology investigations. Various potential biomarkers associated with tumor immunology and immunotherapy have been categorized based on their mechanisms: (1) Genetic markers, including tumor mutation burden, mismatch repair system deficiency, and high microsatellite instability. Next‐generation sequencing technologies can be employed to detect these genetic markers. (2) Surface markers, including PD‐L1, some other inhibitory receptors, and tumor neoantigens. Immunohistochemistry of tumor tissues can be utilized to examine the expression of these surface markers. (3) Cytological markers, including tumor‐infiltrating lymphocytes such as tumor‐infiltrating lymphocytes and exhaustion cells, can be examined by RNA sequencing and immune‐related gene panel scoring. (4) Liquid biopsy markers, including circulating tumor DNA which exists in peripheral blood and can be accessible by blood sampling and analyses. Figure was created with BioRender.com.

Based on their mechanisms, potential biomarkers associated with tumor immunology and immunotherapy can be categorized based on their mechanisms: (1) genetic markers, (2) surface protein markers, (3) TILs, and (4) markers in liquid biopsy (Figure 2 and Table 1).

**TABLE 1:** Different biomarkers utilized in cancer immunology investigations.

Category	Biomarkers	Measurement	Cancer types	Immunotherapies
Surface proteins	PD‐L1	IHC	NSCLC,^34^ RCC,^35^ Melanoma,^35^ etc.	Anti‐PD‐1^35^
	LAG‐3	Flow cytometer, IHC	NSCLC^36^	Anti‐PD‐1^36^
	Tumor neoantigen	Epitope discovery, NGS	Melanoma,^37^ Sarcoma^38^	Anti‐PD‐1,^38^ anti‐CTLA‐4,^37^ ACT^39^
Genetic features	TMB	NGS	NSCLC,^7^ UC,^40^ HNSCC,^41^ etc.	Anti‐PD‐1,^7^ anti‐CTLA‐4^42^
	dMMR/MSI‐H	NGS	CRC,^43^ NSCLC,^44^ GC,^45^ etc.	Anti‐PD‐1,^43^ anti‐CTLA‐4^46^
	DNA methylation	Sulfite sequencing	CRC,^47^ EC,^48^ Melanoma,^49^ etc.	Anti‐PD‐1,^50^ anti‐CTLA‐4^49^
	MHC–TCR axis mutation	Targeted sequencing	NSCLC,^51^ Melanoma^52^	Anti‐PD‐1,^52^ anti‐CTLA‐4^52^
	RNA signatures	RNA sequencing	BC,^53^ NSCLC,^54^ CRC,^55^ etc.	Anti‐PD‐1,^56^ CAR‐T^55^
TILs	CD8^+^ T cells	Flow cytometer	NSCLC,^57^ RCC,^58^ UC,^59^ etc.	Anti‐PD‐1^59^
	CD4^+^ T cells	Flow cytometer	Melanoma,^60^ BC,^61^ GC,^62^ etc.	Anti‐PD‐1,^63^ anti‐CTLA‐4^60^
	Exhausted T cells	Flow cytometer	NSCLC,^64^ Melanoma^65^	Anti‐PD‐1^64^
	B cells	Flow cytometer	Melanoma,^66^ RCC,^67^ BC^68^	Anti‐PD‐1^66^
Liquid biopsy	ctDNA	NGS, sulfite sequencing	NSCLC,^69^ UC,^69^ GC,^69^ etc.	Anti‐PD‐1,^69^ anti‐CTLA‐4^69^
	CTC	Flow cytometer	NSCLC,^70^ OC,^71^ Melanoma^72^	Anti‐PD‐1,^70^ anti‐CTLA‐4^72^
	tdEV	MS, NGS	NSCLC,^73^ Melanoma^74^	Anti‐PD‐1^74^

Abbreviations: ACT, adoptive cell therapy; BC, breast cancer; CAR‐T, chimeric antigen receptor T cell immunotherapy; CRC, colorectal cancer; CTC, circulating tumor cell; ctDNA, circulating tumor DNA; dMMR, mismatch repair system deficiency; EC, esophagus carcinoma; GC, gastric cancer; HNSCC, head and neck squamous cell carcinoma; IHC, immunohistochemistry; MHC, major histocompatibility complex; MS, mass spectrometry.; MSI‐H, high microsatellite instability; NGS, next‐generation sequencing; NSCLC, non‐small cell lung cancer; RCC, renal cell carcinoma; tdEV, tumor‐derived extracellular vesicle; TMB, tumor mutation burden; UC, urothelial cancer.

### 2.1 Surface protein markers

Cancer progression is intricately linked to the immune evasion mechanisms involving immunosuppressive molecule expression.^75^, ^76^ Immune checkpoints, which are expressed on the immune cell surface, are crucial in preventing autoimmunity.^77^, ^78^, ^79^ However, the excessive expression of immune checkpoints leads to immune function suppression.^75^, ^76^ Therefore, ICB therapy effectively hinders tumor growth by obstructing immune checkpoints and enhancing antitumor immune activity.^76^, ^80^, ^81^ ICB development starting from PD‐1/PD‐L1 has brought revolutionary impacts on cancer therapy.^80^, ^81^, ^82^ Nevertheless, only a proportion of patients exhibited disease remission, highlighting the need for individualized ICB utilizing biomarkers.^83^ One approach is to focus on surface protein markers that can be directly detected using immunohistochemistry (IHC).^2^

#### 2.1.1 PD‐L1

PD‐L1 (B7‐H1) exhibits high expression across multiple tumor types and interacts with PD‐1, a crucial immunoregulatory protein found on various immune cell types, thereby facilitating immune evasion by tumors.^84^ ICB employing PD‐1 antibodies specifically target PD‐1, mitigating the immunosuppressive control on T cells and enabling their engagement in tumor cell eradication.^85^ PD‐L1 has been established as the primary biomarker for anti‐PD‐1 treatment, as evidenced by its inclusion in the prescribing information for pembrolizumab.^86^, ^87^, ^88^

Notably, PD‐L1 expression can be influenced by several regulatory mechanisms involving transcriptional and translational levels, which may hinder its clinical applications.^89^, ^90^, ^91^, ^92^ For instance, the JAK–STAT–IRF1 axis severs as a key transcriptional regulator of interferon‐gamma (IFN‐γ) induced PD‐L1 expression,^93^ while translational regulators include PI3K–AKT–mTOR pathway by oncogene activation.^94^ Investigations have revealed mutations in PD‐L1 regulatory pathways that correlate with an unfavorable prognosis after ICB.^51^, ^95^, ^96^ Research into acquired resistance to anti‐PD‐1 ICB uncovered genetic alterations within the interferon and antigen presentation pathways, which have now emerged as crucial biomarkers for predicting relapse following ICB.^97^

It is yet to be investigated how PD‐L1 baseline expression affects tumor progress in the early stages of the disease. A recent study in 2022 demonstrated that all patients of untreated stage II or III triple‐negative breast cancer (TNBC) exhibited improved levels of pathological complete responses, regardless of PD‐L1 expression.^9^ In contrast, in the KEYNOTE‐355 trial, pembrolizumab plus chemotherapy led to prolonged event‐free survival among metastatic TNBC patients with a high PD‐L1 expression level.^98^ Similarly, atezolizumab therapy efficiency was only associated with high PD‐L1 level in late‐stage metastatic patients instead of early‐stage patients.^99^, ^100^ In conclusion, there is only insufficient evidence for the prediction efficiency of PD‐L1 expression in nonmetastatic tumors.

#### 2.1.2 Other surface markers

Novel immunotherapy targets and immune biomarkers are of high interest. CTLA‐4 is a transmembrane protein expressed in activated CD4+ and CD8+ T cells, which suppresses effector T cells as an early IC in immune priming.^101^ Biomarker studies of anti‐CTLA4 therapies focused on the diversity of peripheral blood lymphocytes (PBLs) rather than tumor cells.^102^ In various tumor types, increased expression of the T‐cell costimulatory molecule (ICOS) on PBLs and TILs has been observed after CTLA4 blockade, suggesting that ICOS on immune cells may serve as a potential biomarker for anti‐CTLA‐4 therapy.^103^

Other targets and surface markers include LAG3, TIM3, B7H3, NR2F6, TIGIT, VISTA, and BTLA.^2^, ^104^ High expression levels of TIM‐3 on TILs have been negatively correlated with OS.^105^ In NSCLC, coexpression of PD‐1, LAG‐3, and TIM‐3 after anti‐PD‐1 treatment was significantly associated with significant T cell suppression and shorter OS.^36^ In a study across several cancer types, multiplexed IHC demonstrated a higher prediction accuracy for PD‐1 blockage when compared with other biomarkers including PD‐L1 expression, TMB, and gene expression signatures.^106^ Analyses for immunotherapy‐treated NSCLC cases revealed CD56 and CD4 expression in the CD45^+^ compartment to be an efficient biomarker for several clinical outcomes.^107^ As for the tumor compartment, the CD44 expression in the tumor cells serves as a novel prognostic factor for extended PFS and OS under anti‐PD‐1 treatment.^108^ Despite associations, these emerging surface markers still lack robustness for clinical use.

#### 2.1.3 Tumor neoantigen

Neoantigens, as unique proteins expressed exclusively in tumor cells targeted by T cells in the immune system, have the potential to serve as ideal biomarkers for ICB including anti‐PD‐1^38^ and anti‐CTLA‐4.^37^ During tumor development, nonsynonymous mutations occur, leading to alterations in the amino acid coding sequence and the production of abnormal proteins specific to the tumor. These abnormal proteins can activate the immune system, triggering an immune response against the tumor.^109^ The presence of neoantigens with high affinity for major histocompatibility complex (MHC) increases the likelihood of an effective immune response.^110^ However, assessing the “quality” of neoantigens, which refers to their ability to induce immune recognition and activation, remains a challenge.^111^ The current measurement approach, known as epitope discovery, can be achieved through two main approaches. The first method involves detecting mutations in the exons and subsequent candidate screening using MHC binding assays.^112^ Alternatively, neoantigens can be directly obtained by TCR sequencing of tumor‐reactive T cells.^38^ Currently, neoantigens are primarily used in conjunction with other biomarkers, such as TMB. The direct utilization of neoantigens as biomarkers requires the development of assays and algorithms capable of accurately detecting both the quantity and quality of neoantigens within a tumor.^113^

#### 2.1.4 Conclusions for cell surface markers

Surface markers, including PD‐L1 and other immune checkpoints as well as neoantigens, play a crucial role in cancer immunology investigations.^86^, ^88^, ^105^ PD‐L1 serves as the only biomarker approved by clinical guidelines for ICB, but challenges remain due to its complex regulatory mechanisms and sufficient evidence in nonmetastatic cancer.^9^, ^87^, ^97^ Besides PD‐L1, other surface markers including neoantigens have also shown potential as biomarkers for immunotherapy but require further validations.^36^

### 2.2 Genetic features

The advancements in sequencing technologies, including polymerase chain reaction and next‐generation sequencing (NGS), have provided a crucial foundation for the research and application of genetic biomarkers.^114^, ^115^, ^116^ Commonly used genetic markers include defective mismatch repair (dMMR)/MSI‐H (high microsatellite instability) and TMB, both associated with mutational loads. The abundance of mutations increases the likelihood of self‐neoantigens being immunogenic, leading to the activation of T cell responses.^117^ Similar genetic markers include somatic copy number variation which has a larger‐scale impact on genome structure and has been reported to have prognosis predictive value in CRC.^118^, ^119^

#### 2.2.1 Tumor mutation burden

TMB refers to the total number of mutations detected per megabase and serves as a prominent biomarker for ICB.^120^, ^121^, ^122^ This assertion is based on the assumption that an elevated presence of mutant proteins will generate immunoreactive neoantigens, enhancing immunogenicity.^122^, ^123^ However, recognizable tumor neoantigens can occur even in a low mutation setting, and a high number of mutations does not guarantee the presence of immunogenic neo‐antigens.^124^ Meanwhile, the complex microenvironment influences T cell‐mediated tumor killing and may compromise the inflammatory microenvironment .^125^ Therefore, cancer immunology investigations concerning TMB must be considered along with multiple other factors including microenvironment features and specific mutation panels.

Accumulating evidence has suggested that associations between TMB and immunotherapy differ between different cancer types.^12^, ^114^ An observation study covering 32 cancer types compared the predictive efficiency of TMB‐H on ICB treatment.^12^ TMB‐H demonstrated significantly better survival in tumors whose neoantigen loads positively correlated with CD8^+^ T cell levels, including lung cancer and melanoma. However, for cancer where neoantigen did not positively correlate with CD8^+^ T cell levels, such as breast and prostate cancer, TMB‐H tumors failed to achieve better outcomes.^12^ A possible explanation is that the predictive efficacy of TMB‐H predominantly relies on the basal immune cell infiltration level.

Different tumor types depend on different mutational events during development. Thus, TMB across tumors cannot be defined by one universal mutational signature.^117^ TMB characteristics should be more accurately identified for a certain cancer type or a given immunotherapy.^126^, ^127^ Signatures linked to external mutagens such as ultraviolet radiation and smoking are more prevalent in melanoma and lung cancer. In contrast, signatures associated with deficiencies in DNA repair genes (*MRC1, POLE*) are more prominent in endometrial, colorectal, and esophagogastric cancers.^128^

#### 2.2.2 Mismatch repair system and MSI

Microsatellites encompass brief recurring sequences of one to six nucleotides dispersed across the genome and are notably susceptible to DNA mismatch during replication.^129^, ^130^, ^131^ In normal tissues, the crucial mismatch repair (MMR) system typically rectifies these errors in DNA replication or recombination.^132^, ^133^ As a result of MMR gene mutations, there is an exponential increase in mutation probability in microsatellite genome regions, causing high‐frequency MSI.^134^, ^135^, ^136^ dMMR/MSI‐H has been considered a key prognosis influencing factor for CRC.^43^ For ICB, dMMR/MSI‐H tumors have been reported to benefit from PD‐1 antibody treatment.^44^, ^137^ In 2017, pembrolizumab was approved by the United States Food and Drug Administration for treating relapsed or refractory solid tumors with MSI‐H and dMMR, marking the first approval of a biomarker that is agnostic to tumor‐type.^45^, ^137^, ^138^

Notably, the impact of indel mutations varies according to the microsatellite location.^139^ When indel occurs within noncoding segments, little effect can be observed. However, indel mutations in regulatory, splicing, or protein‐coding regions contribute to frameshifts which likely yield immunogenic neoantigens.^139^ About 83.3% of the MSI‐H tumors demonstrated a high level of TMB, while 16% of the TMB‐H tumors are MSI‐H, suggesting that MSI‐H may serve as a contributing factor of TMB‐H.^140^ However, as evidence does exist for the ICB treatment of certain hypermutator cancer types, especially for CRC patients with a high level of MSI,^141^, ^142^ there is still uncertainty about what threshold should be applied for treating different cancer types with ICBs.^121^, ^143^, ^144^

#### 2.2.3 DNA methylation

Associations exist between epigenetic characteristics and other biomarkers, suggesting intricated underlying mechanisms. In NSCLC, direct links between methylome alterations and TMB were observed.^145^ In breast cancer, *NEFM* promoter hypomethylation was reported to be associated with increased immune infiltration and plays a role in TME reshaping.^146^ Besides, methylations serve as targets for epigenetic therapy. Thus, identifying epigenetic biomarkers helps the combined application of epigenetic therapy and immunotherapy.^147^

Various cancer subtypes exist with distinct progression and immunologic patterns based on the epigenetic landscape. For example, CRC can be categorized into MSI, chromosomal instability, and CpG island methylator phenotype (CIMP), in which CIMP is characterized by hypermethylation of CpG sites around certain gene promoter regions.^47^ By calculating the scores of a series of key gene phenotypes within the CIMP subtype, predictions can be made regarding the therapeutic prognosis of patients.^47^, ^148^, ^149^ Besides, ICB response prediction models utilizing DNA methylation profiles have emerged for several cancer types including melanoma,^150^ esophagus carcinoma,^48^ NSCLC,^151^ glioma,^152^ and bladder cancer.^153^

However, despite the potential of DNA methylation being a reliable biomarker in various cancers, fundamental exploration is still needed. One key gap lies in the fact that in vivo methylation exists in a balance between generation and removal, while measurements only reflect stable levels instead of turnover rates. Therefore, dynamic analysis of tumor DNA methylation in tumor immunity is crucial for a comprehensive understanding.^154^

#### 2.2.4 Genomic alterations in specific genes

A variety of alterations in specific genes including the MHC–TCR axis and PD‐L1/*CD274* gene serve as biomarkers individually. MHC diversity affects neoantigen presentation, while TCR repertoire determines antigen recognition.^52^, ^155^ Higher MHC heterozygosity or TCR clonality indicates the presence of tumor‐reactive T‐cells and correlates with improved survival during ICB treatment.^155^, ^156^ Mutation in *POLE/POLD1* results in the heightened hydrophobicity of TCR‐contact residues and thus enhances T‐cell recognition and interaction.^157^, ^158^ However, the relationship between TCR clonality and ICB response differs depending on different checkpoint blockages and this phenomenon is partly explained by different associations between TCR mutation, neoantigen load, and TILs.^159^, ^160^ Apart from MHC–TCR axis, various genomic alterations can independently influence immunotherapy response, such as PD‐L1/*CD274* gene amplification and *B2M* mutations.^88^, ^161^, ^162^, ^163^

#### 2.2.5 RNA signatures

With the progress of sequencing techniques and comprehensive databases, identifications of RNA signatures using transcriptomic RNA data have become a prevalent practice.^164^ Examples of transcriptomic signatures include MHC class II (HLA‐DR) expression in melanoma,^56^ prognostic hypoxia‐immune genes in TNBC,^53^ and ferroptosis signatures in breast cancer.^165^ Furthermore, mRNA post‐transcriptional modifications have been reported to be associated with tumor immunology.^166^, ^167^, ^168^ Additionally, noncoding RNA (ncRNA) has gained significant attention as a novel biomarker,^169^, ^170^, ^171^, ^172^ including lncRNA,^173^ circRNA,^54^ and miRNA.^55^

#### 2.2.6 Conclusions for genetic features

The TMB, dMMR/MSI‐H, DNA methylation, alterations in specific genes, and RNA signatures have shown significant implications in cancer immunology.^47^, ^120^, ^138^ However, TMB and MSI may not apply to all cancer types and the specific threshold for different cancer types remains unclear.^12^, ^114^ DNA methylation patterns can influence immune responses and tumor progression but still need further elucidations.^154^

### 2.3 Tumor‐infiltrating lymphocytes

Interaction, activation, and costimulation of lymphocytes are essential for a successful antitumor immune response, including CD8^+^ T cells, CD4^+^ T cells, and B cells.^11^ The presence and proportion of different TIL subgroups, as well as their functional stage, differentiation process, and composition structure, have a fundamental impact on tumor immunotherapy. For instance, the T cell‐inflamed gene expression profile (GEP) has been reported to correlate with a good ICB prognosis in several cancer types.^118^, ^174^, ^175^ This GEP contains IFN‐γ‐responsive genes related to antigen presentation, chemokine expression, and adaptive immunity and serves as a quantification of T cell‐inflamed microenvironment which can improve response to anti‐PD‐1 treatments.^176^ Besides, the tertiary lymphoid structures (TLSs) as de novo lymphoid tissue resembling lymphoid organ structure serves as a promising prognostic predictor for improved post‐treatment survival.^66^, ^67^

#### 2.3.1 CD8^+^ tumor‐infiltrating T cells

CD8^+^ T cells are characterized by their antitumor functions and are also referred to as cytotoxic T lymphocytes (CTLs) which serve as a producer for high levels of cytotoxic molecules (such as granzyme) and antitumor cytokines (such as tumor necrosis factor‐α, TNFα). Studies have demonstrated that CTLs are linked to favorable prognoses across diverse cancer types. Under physiological conditions, CTLs are transformed into memory subtypes to preserve long‐term protection capacity. Notably, memory CD8^+^ T cells are a heterogeneous group that can be further classified into central memory T (T_CM_), effector memory T (T_EM_), and other subgroups including stem cell‐like memory T (T_SCM_), and effector memory RA^+^ T (T_EMRA_) cells.^177^, ^178^ Despite naïve‐like features and limited direct effector functions, T_CM_ have been reported to characterize ICB responders in naive tumors.^179^ It has been demonstrated that treatment of T_CM_ cells with ICB induces a cytolytic gene signature and an effector‐like phenotype. Increased expression of LAG3, BTLA4, and PD‐1 were observed in nonresponders.^180^ T_EM_ cells exhibit proinflammatory functions and serve as an independent prognostic factor of OS.^181^ Recently, peripheral tissue‐resident memory (T_RM_) CTLs which normally stay in peripheral tissues and can be recruited for antitumor immune responses have attracted attention.^182^ CD103^+^ T_RM_ was associated with a favored prognosis, and there was also an increased T_RM_ abundance in ICB responders.^183^

#### 2.3.2 CD4^+^ tumor‐infiltrating T cells

CD4^+^ T cells function with CD8^+^ T cells in antitumor immune response.^184^ Among CD4 T cells, conventional helper CD4^+^ T cells (T_HC_) use CD40L on the cell surface to interact with CD40 on dendritic cells (DCs) and help CD8^+^ T cells in the priming process.^184^ Notably, only T_HC_ cells with functioning MHC‐I and MHC‐II (instead of a single MHC‐I) were able to eliminate tumors after ICB.^185^ Additionally, the quality of T cell response is greatly influenced by the diversity and specificity of TCR. Studies have shown that the expansion of T cell clones can be observed in both ICB responders and nonresponders.^63^ A higher TCR clonality and diversity correlated with improved response to ICB, as confirmed by multi‐variate regression models in a randomized controlled trial.^60^

Unlike CD8^+^ T cells and previously mentioned CD4^+^ subgroups, CD4^+^ regulatory T (T_reg_) cells characterized by the high expression of CD25 and FOXP3 suppress CD8^+^ T cells by counteracting tumor immune response.^186^ T_reg_ cells have been associated with poorer survival in many kinds of solid tumors including breast, gastric, pancreatic, colon, and cervical cancers,^61^, ^62^, ^187^, ^188^ while T_reg_ depletion contributes to the success of anti‐CTLA‐4 treatments.^189^ Hyperprogressive disease (HPD) is a rare event in which a rapid type of tumor is caused by ICB. A recent study demonstrated that increased proliferation of T_reg_ can be observed in HPD patients, and T_reg_ cell depletion may serve as an HPD prevention before anti‐PD‐1 therapy.^190^ Notably, recent studies have revealed diverse subgroups within Tregs. For instance, Treg subgroups expressing GZMB, LAG3, TIM3, or CCR8 exhibit significant immunosuppressive activity in CRC,^191^ while CD30^+^OX40^+^ Tregs serve as negative Treg regulators, correlating with improved prognosis.^192^ However, the significance of these diverse Treg subgroups as immunotherapy biomarkers remains to be explored.

#### 2.3.3 Exhausted T cells

T cell exhaustion refers to the dysfunction of T cells caused by prolonged exposure to antigens. It is characterized by reduced cytolytic and proliferative ability, accompanied by elevated expression of various surface receptors such as PD‐1, CD103, CX3CR1, CD39, and TIM3.^178^, ^193^ Changing T cell phenotype is critical for switching between an inflammatory immune response that inhibits tumor growth or a regulatory state that promotes tumor growth.^178^

Exhausted CD8^+^ T cells can be further classified into progenitor stem‐like exhausted (T_PE_) cells and terminally exhausted T (T_EX_) cells.^65^ T_PE_ cells possess expressions of transcription factor T cell factor 1 (TCF1) and are characterized by maintained tumor antigen‐specific immune response capacity.^194^ This subgroup of exhausted T cells will eventually differentiate in the ultimate T_EX_ stage with a decrease in TCF1 expression.^194^ A single‐cell transcriptional analysis in 2018 have shown that PD‐1^high^ CD8^+^ T cells serve as a predictive biomarker for anti‐PD‐1 therapy in NSCLC as anti‐PD‐1 therapy impact on T_PE_ cells instead of T_EX_ cells.^64^ Several other studies also support this conclusion, showing that T_PE_ cells increased their cytotoxic capacity while T_EX_ cells did not show a response to ICB therapy.^65^, ^194^, ^195^

#### 2.3.4 Tumor‐infiltrating B cells

Studies have shown that TIL B cells were associated with an improved immunotherapy response as well as better survival.^66^, ^196^, ^197^ For instance, melanoma and renal cell carcinoma (RCC) samples with a higher expression level of B cell gene panels had a higher response rate to ICB treatment. ICB responders exhibited an increased memory B cell proportion, an enhanced BCR diversity, and a larger B cell clonal expansion.^67^ The coexistence of B cells and T cells facilitated the formation of TLS, served a curial role in TME formation in melanoma, and led to an improved prognosis after cancer immunotherapy.^66^ Notably, ICOSL^+^ B cells, a small subgroup of TIL B cells, were found to serve as an antitumor immune response booster after neoadjuvant chemotherapy in breast cancer.^198^ Another subgroup of TIL B cells, regulatory B (B_reg_) cells, have been reported to play an important regulatory role in cancer immunity.^199^, ^200^, ^201^, ^202^ However, evidence on the role of B_reg_ in human in vivo studies is still limited.^202^

#### 2.3.5 Conclusions for TILs

TILs play a pivotal role in the tumor immune microenvironment, reflecting the complex interplay between the immune system and the tumor.^11^ CD4^+^ T cells assist in activating other immune cells, while CD8^+^ T cells directly recognize and eliminate tumor cells.^179^, ^184^ Exhausted T cells are characterized by sustained antigen exposure and functional impairment. Among them, the T_PE_ subgroup retains responsiveness to immunotherapy and partially explains the success of ICB.^64^ Additionally, TIL B cells are attracting more and more attention as a key factor associated with an improved immunotherapy response.^67^

### 2.4 Liquid biopsy

Liquid biopsy involves the analysis of circulating tumor material mostly in blood and has become one of the most powerful tools in the management of various kinds of cancer.^203^, ^204^, ^205^, ^206^ Compared with invasive tissue biopsy, noninvasive liquid biopsy provides a deeper understanding of cancer dynamics by utilizing frequent analysis of circulating biomarkers including circulating tumor DNA (ctDNA), circulating tumor cells (CTCs), and extracellular vesicles (EVs).^207^

#### 2.4.1 Circulating tumor DNA

ctDNA is released from necrosis/apoptotic tumor cells into the bloodstream.^120^ By identifying genetic mutations in ctDNA, it becomes possible to gain real‐time insights into the tumor states.^69^, ^208^, ^209^ ctDNA pool provides a greater accuracy for determining TMB since it represents mutations from multiple tumor subclones.^210^, ^211^ Estimates of TMB based on blood samples (bTMB) exhibited significant concordance with tissue TMB (tTMB) and serves as a predictive biomarker in ICB response.^69^, ^211^, ^212^ Similarly, estimations of MSI based on blood samples (bMSI) can be used for ICB response prediction as well.^213^, ^214^, ^215^ While these findings recommended ctDNA‐based mutation estimations in patients whose tissue biopsy cannot be easily obtained, further studies concerning a broader range of cancer types and patients with low ctDNA levels are still needed.^208^

Aside from TMB and MSI, longitudinal ctDNA tracings can provide insights into tumor dynamics and tumor immunity monitoring.^209^, ^216^ The CheckMate‐816 trial focusing on neoadjuvant ICB of NSCLC patients revealed a correlation between the elimination of specialized ctDNA panels and pathological complete response.^216^ Post‐treatment ctDNA detection can also help identify patients with higher risks of cancer relapses, even only 3 days after operations.^217^, ^218^

Recently, DNA methylation in ctDNA has been shown to serve as novel diagnostic and prognostic biomarkers.^219^, ^220^ The aberrant DNA methylation status of ctDNA has emerged as a promising biomarker for the prediction of drug response in several cancer types including lung cancer, breast cancer, CRC, and prostate cancer.^219^, ^221^ Further research is needed to elucidate the functional significance of ctDNA methylation and its relationship with TME. Besides, exploring the dynamic of ctDNA methylation will also contribute to a deeper understanding of tumor‐immune interactions.

#### 2.4.2 Circulating tumor cells

CTCs are tumor cells that have spread from primary or metastatic tumors to blood and serve as an intermediate stage in the metastasis process.^222^ The tumor immunological characteristics of CTCs are closely associated with their ability to evade immune surveillance.^223^ Despite thousands of tumor cells entering the bloodstream daily, only a small fraction of CTCs can be detected due to the loss of protective immunosuppressive microenvironment in the primary tumor.^223^ CTCs can promote the formation of an immunosuppressive environment by downregulating MHC‐I molecules and upregulating immune checkpoints such as PD‐L1.^224^ Therefore, higher levels of CTCs may indicate stronger immune suppression and lower immune cell activity.^225^

Multiple studies have shown that CTC counts can serve as prognostic indicators for patients, despite their low detection rate. Baseline CTC counts have been employed as prognostic markers, and correlate with patients exhibiting either long or short OS.^70^, ^226^, ^227^ For immunotherapies, several studies have revealed its predictive role. A study on metastatic or relapsed NSCLC immunotherapy candidates revealed a significant positive correlation between an extensive mutation burden and a higher number of CTCs.^228^ Another study further demonstrated that CTCs PD‐L1 expression can efficiently predict ICB treatment outcomes advanced NSCLC.^229^

In conclusion, the value of CTCs as biomarkers lies primarily in their quantity and biological characteristics. Currently, the relationship and molecular mechanisms between CTC formation and their immune evasion ability are not yet fully understood. Further research advancements in this area will facilitate the application of CTCs as biomarkers for cancer immunology and immunotherapy. Noteworthy, for CTC to be used for molecular diagnosis, the purity of CTCs is an important influencing factor on prediction efficiency. Therefore, the development of microfluid chip, nanotechnology, and 3D bio‐printing plays a crucial role in improving CTC capture efficiency and purity.^227^, ^230^

#### 2.4.3 Extracellular vesicles

EVs, membrane‐bound vesicles secreted by various cell types, have been identified and isolated from different bodily fluids, providing a noninvasive approach to characterize the originating tumor cells.^231^ Tumor‐derived EVs (tdEVs) carry a wide variety of tumor neoantigens and exhibit a distinct molecular signature that mirrors the genetic complexity.^232^ tdEVs have emerged as potential mediators of cellular communication and modulators of TME, particularly in the establishment of immunosuppressive environments in distant metastatic sites.^233^, ^234^ TDEs have been identified as immunoregulatory factors that modulate various immune and stromal cells, including T cells,^235^ T_reg_ cells,^236^ and cancer‐associated fibroblasts (CAFs).^237^

tdEVs serve as carriers of diverse cargo, offering valuable insights into individualized tumor status.^238^ tdEVs containing DNA enable the identification of genetic mutations, providing information about cancer‐specific alterations.^239^ Additionally, tdEVs capable of predicting individualized treatment responses have been identified.^74^, ^240^ Significant differences in exosomal PD‐L1 levels before treatment have been observed between responder and nonresponder, suggesting that exosomal PD‐L1 is associated with anti‐PD‐1 responses.^74^ However, due to its low content, even minimal contamination can lead to lowered efficacy, necessitating the development of accurate detection methods.^238^

#### 2.4.4 Conclusion for markers in liquid biopsy

Biomarkers in liquid biopsy, including ctDNA, CTCs, and tdEVs, have shown significant relevance in cancer immunology investigation due to their shared advantages of enabling noninvasive real‐time monitoring of tumor dynamics.^207^ They provide an accessible approach for longitudinal assessment of cancer progression.^217^, ^218^ However, challenges remain due to the low abundance of these biomarkers in the bloodstream.^238^ The development of biomedical engineering technologies, particularly microfluidic chips, holds promise for advancing this field by improving sensitivity, efficiency, and reliability in liquid biopsy.^227^, ^230^

## 3 IN VITRO PRECLINICAL MODEL FOR TUMOR IMMUNOLOGY INVESTIGATION

Rapid development of immunotherapy necessitates convenient, stable, and cost‐effective in vitro models for cancer immunology investigations.^18^ Additionally, tumor immunology research requires effective preclinical models that faithfully reproduce the in vivo tumor, particularly the tumor‐immune interactions.^241^ These dual demands have spurred the development of preclinical in vitro models effectively recapitulating the native TME.^242^ Successful TME replication involves two key aspects: (1) reproduction of cellular components, including tumor components, immune cell subsets, and stromal cell subsets^243^, ^244^; (2) preservation of cellular functions, including the cytotoxicity of T and natural killing (NK) cells, antibody production by B cells, antigen presentation by myeloid cells, and ECM remodeling by CAFs.^28^, ^242^ Traditional 2D culture models have limitations in effectively replicating TME due to reasons including flat monolayer configurations and less representative distributions of oncogenes and tumor suppressors.^21^, ^23^ Compared with 2D models, 3D culture models create polarization of cells with distinct basal and apical poles through suspension, embedding, or advanced chip structures. Alterations in tissue microstructure result in modified distributions of oxygen, nutrients, and metabolites and further lead to optimized genomic and protein characteristics.^15^, ^18^, ^245^ Additionally, by crosslinking biological materials in a manner that mimics in vivo tissue, 3D cultures create a solid ECM that closely resembles the properties of real TME.^246^ This allows for the simulation of mechanical interactions between cells and the ECM, which are essential for various biological processes such as tumor growth, adhesion, migration, and immune infiltration.^247^

### 3.1 3D in vitro culture

**FIGURE 3:**
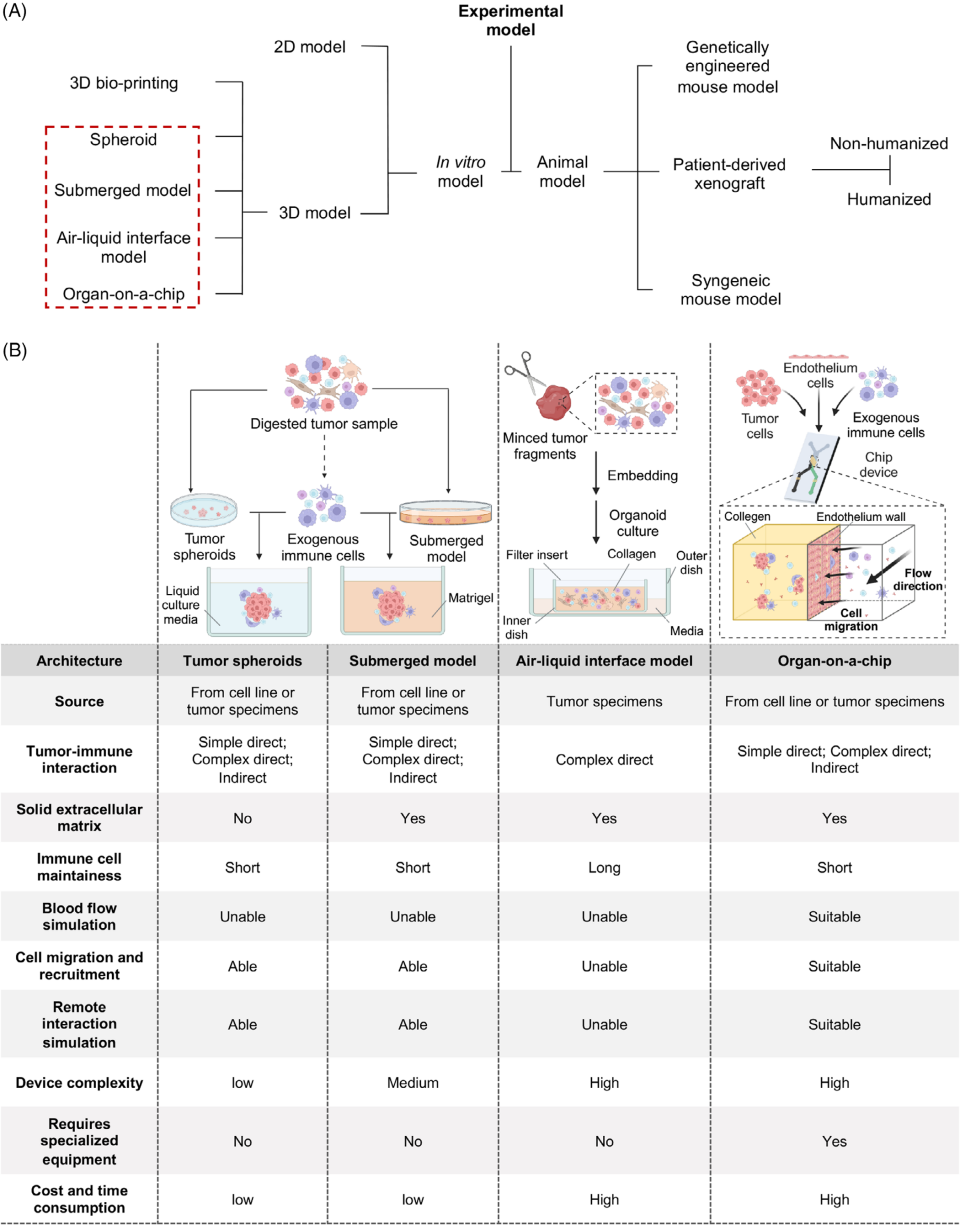
Comprehensive comparison of 3D tumor‐immune coculture system with different construction approaches. (A) Experimental models can be classified into in vitro models and animal models. In vitro models can be further divided into 2D models and 3D models which encompass spheroid, submerged model, air–liquid interface (ALI) model, and 3D bio‐printing based model. For animal models, they can be separated into syngeneic models, genetically engineered models, and patient‐derived xenografts. (B) Tumor immune microenvironment can be generated in in vitro 3D tumor‐immune coculture by four construction approaches. (1) Left: for tumor spheroid and submerged model, tumor‐immune interaction can be simulated by adding exogenous immune cells. (2) Middle: in the ALI model, minced tumor tissue fragments containing both tumor and immune cells are embedded in collagen. (3) Right: for the microfluid chip, tumor cell spheroids are mixed with collagen and injected into the central compartment while immune cells are circulating. Figure was created with BioRender.com.

3D tumor‐immune coculture systems can be divided into several subtypes based on construction architectures. The construction approach determines the specific spatial distribution of cancer and immune cells, thus having a fundamental impact on the characteristics of the coculture system (Figure 3A).

#### 3.1.1 Spheroid

Spheroids are collections of cells growing in three dimensions while suspended with or without an ECM.^18^ A more complex model, such as a submerged model or microfluid chip, can be built based on tumor spheroid.^28^, ^248^

A variety of tumor types have completed the construction of 3D spheroid models, such as lung cancer,^243^ prostate cancer,^249^ and head/neck squamous cell carcinoma (HNSCC).^250^ Generally, tumor spheroid exclusively preserves tumor epithelium cells. But with gentle digestion, spheroid can retain the immune cell components within the primary tumor for a period and preserve responsiveness towards immunotherapy.^243^

Spheroids are used for testing immunotherapies by adding exogenous immune components, mainly to evaluate the effectiveness of therapeutic antibodies and conduct drug screening for improving immune cell infiltration and antitumor effects against the targets of spheroids.^249^, ^251^, ^252^ Immune cells that can be added include CTLs,^249^, ^253^ Vδ2T cells,^251^ and NK cells.^252^

Overall, the spheroid model is a critical step forward in moving from 2D to 3D culture with the lowest cost and build difficulty. Despite the lack of retention of immune components, it is still one of the most widely used 3D tumor‐immune coculture models.

#### 3.1.2 Submerged model

Submerged model (Figure 3B) cultures tumor spheroids with exogenously added stromal or immune cells in Matrigel submerged in a culture medium.^254^ It is widely used and has been built for different cancer types, including breast cancer,^255^, ^256^ lung cancer,^257^, ^258^ ovarian cancer,^259^ colon cancer,^241^, ^257^ pancreatic cancer,^260^, ^261^ and gastric cancer.^248^ In the submerged model, native stroma cells and immune components cannot be preserved conventionally. Exogeneous cell types include stromal cells like CAFs,^258^ and immune cells like CTLs,^261^, ^262^ tumor‐associated macrophages (TAMs),^261^ and PBLs.^258^, ^263^

If organoids can be constructed suitably and a short time after tumor sample collection, part of the original immune cell components and even the original immunosuppressive environment in the tumor can be preserved.^241^, ^259^ To better preserve original tumor components, the time interval between sample collection and organoid construction must be possibly short (often less than one day), and a softer digestion method should be used during organoid construction.^259^ Submerged models utilizing patient tumor samples from high‐grade serous ovarian cancer were employed to investigate the efficacy of dual‐specific anti‐PD‐1/PD‐L1 antibodies.^259^ By adding ICBs and IL‐2, activation of T cells and NK cells can be observed.^259^

Submerged models have provided insights into generating and screening highly cytotoxic and tumor‐selective lymphocytes.^257^, ^264^ Tumor‐reactive T cells with high cytotoxicity and specificity from peripheral blood can be efficiently enriched in coculture systems of autologous patient‐derived organoids (PDOs) from patients with dMMR CRC and NSCLC.^257^ Submerged models can also reproduce characteristic responses of immunotherapy and have been widely used as a model for immunotherapy testing, including ICB,^241^, ^256^ Bacille Calmette‐Guerin (BCG) immunotherapy,^263^ High‐affinity neoantigens,^265^ iRGD peptide,^253^ Bi‐mab antibody,^266^ Chimeric antigen receptor T cell immunotherapy (CAR‐T),^255^, ^267^, ^268^ cibisatamab,^269^ and Vδ2T.^270^ For instance, the cytotoxicity of peripheral blood mononuclear cells (PBMCs) activated with BCG vaccine were validated in 3D coculture systems involving HNSCC cell line FaDu.^263^

#### 3.1.3 Air–liquid interface model

In the ALI model, minced patient‐derived tumor tissues are directly embedded in the collagen matrix without digestion (Figure 3B). Collagen‐embedded tumor tissue is placed inside the inner plate where the culture medium can diffuse through the permeable plate wall from the outer plate.^271^ The inner plate is in direct contact with the air to ensure adequate oxygen supply to the organoid. As the ALI model uses patient‐derived tumor tissue *en bloc* that includes endogenous stromal and immune cells, it has a strong advantage in simulating the native TME.^244^ Tumors from multiple sources, for instance, skin, colon, pancreas, and lung can successfully prepare and form ALI models with high reproducibility.^242^, ^244^ IHC and fluorescence staining showed that ALI cultures not only effectively preserve stromal components such as fibroblasts and myofibroblasts but also retain a wide range of immune components including CD8^+^ and CD4^+^ T cells, B cells, NK cells, and PD‐1^+^ CD3+ T cells. Further single‐cell RNA sequencing (scRNAseq) revealed that 85% of T and B cells in ALI can be detected using VDJ enrichment assays, enabling the linkage of cell‐type identification and immune repertoires from the same cells.^242^ Compared with the submerged model, the ALI model greatly improved lymphocyte lifespan (1 month without IL‐2, and 60 days after IL‐2 was added).^242^ ALI model can also reproduce the TCR repertories of the original tumor and can be used for the tests of immunotherapy.^242^, ^244^

Among all constructs, the ALI model is the only one that does not require digestion and uses tissue *en bloc* directly for model construction. This construction mode directly preserves the original cellular components in the tumor tissue and greatly extends the survival time of the nonmalignant components. However, the culture method relying on fresh tumor tissue slices also limits the ability of ALI models to incorporate exogenous immune components from the external for TME reconstruction. Additionally, ALI models cannot be constructed using tumor cell lines other than fresh tissue.

#### 3.1.4 Organ‐on‐a‐chip

Due to breakthroughs in biomedical engineering and material chemistry, the use of microfluidic chip technology in tumors has made great progress.^272^ Tumor tissues are minced, digested, sieved to collect spheroids, mixed with collagen, and injected into the central gel compartment of the microfluid chip.^272^ (Figure 3B) The culture medium is added to the fluid channel. Microfluidic chips have been widely used in the construction of in vitro 3D tumor‐immune models,^273^, ^274^, ^275^, ^276^ and have been used to test the efficacies of various immunotherapies such as ICB,^28^ CAR‐T,^275^, ^277^ and Vδ2T.^270^ Utilizing HeLa and NK‐92 cell line, rail‐based microfluidic design was integrated within a single 96‐well to achieve high‐throughput 3D coculture of cytotoxic lymphocytes with cancer cells.^275^ Besides, combining the PDO with the spheroid‐based microfluid chip can also effectively preserve the original immune components in tumor samples.^28^ The tumor organotypic slice model is a special type of organ‐on‐a‐chip. It allows for the investigation of native TME by directly utilizing undigested tumor tissue slices, such as exploring the role of astrocytes and microglia in immunosuppressive environment formation in glioblastoma.^278^

Microfluidic chips can simulate complex TMEs including blood vessels,^279^ and the blood–brain barrier (BBB).^276^ In the vascular simulation, tumor cells, endothelial cells, and fibroblasts were cocultured in a certain proportion in the collagen‐embedded microfluidic chip. The cell mixture could spontaneously form vascular structures composed of endothelium cells.^273^, ^279^ To simulate the BBB, microfluidic chips can be utilized to coculture patient‐derived breast cancer cells, endothelial cells, and astrocytes. This in vitro model accurately replicates BBB structure and allows for the investigation of tumor‐immune interactions and their impact on tumor‐brain metastasis.^276^ Microfluidic chips can also generate dynamic gradients of various substances. Therefore, it can be used as a cell migration model,^270^ or a tumor‐lymph node remote interaction model.^274^

Collectively, microfluidic chips are capable of simulating complex environments like blood circulation and cellular barriers.^273^, ^276^ However, due to its size limitation on spheroids, it only has a limited effect on the reconstruction of histological morphology.

#### 3.1.5 Conclusions for different 3D in vitro model architectures

Different 3D in vitro culture architectures, including spheroids, submerged models, ALI models, and organ‐on‐a‐chip systems, offer unique advantages and applications in cancer immunology investigation (Figure 3B). Spheroids as the earliest tumor 3D in vitro culture represent a transition from 2D to 3D but lack the preservation of immune components within TME.^249^, ^251^, ^252^ Submerged models provide a solid noncellular ECM and offer versatility in studying various aspects of cancer immunology.^241^, ^259^ ALI models exhibit the strongest capability to preserve the native TME but are limited to tissue culture and encounter difficulties incorporating exogenous immune components.^242^, ^244^ Organ‐on‐a‐chip systems excel in simulating complex environments and interactions. However, the limited sample size, high costs, and model complexity hinder their wider application.^273^, ^274^, ^275^, ^276^

### 3.2 3D bio‐printing technology for in vitro model construction

The realm of tissue engineering witnessed the rise of 3D bio‐printing as a highly promising methodology for fabricating intricate biological structures.^280^ As for tumor immunology, 3D bio‐printing is gaining prominence as a powerful tool due to its ability to preserve tumor cells in a near‐native state. It has found extensive applications in oncology research, providing new avenues for studying tumor immunology.^280^, ^281^

#### 3.2.1 Approaches of 3D bio‐printing

The field of 3D bio‐printing relies on three key technological approaches: biomimicry, autonomous self‐assembly, and mini‐tissues. These enable the printed in vitro tumor model to exhibit the envisioned functions and structures, closely resembling primary tumors.^282^ 3D bio‐printing technologies used in tumor‐immune coculture include inkjet, laser‐assisted bio‐printing (LAB), and extrusion‐based bio‐printing (EBB).

Inkjet bio‐printers precisely deposit bio‐ink onto the targeted printing surface using thermal or acoustic mechanisms, ensuring a continuous flow or controlled droplet release from the nozzle.^283^, ^284^ With the ease of modification, low cost, and fast speed, it has been most widely used for cancer immunology studies.^285^ LAB uses laser‐induced forward transfer, in which high‐pressure bubbles are generated by a high‐energy laser pulse in a thin biomaterial layer, ejecting it onto a specific area.^286^ This precise process enables accurate biomaterial deposition, making LAB a promising technique for complex tissue fabrication in TME reconstructions.^287^ LAB offers the advantages of accommodating a wide range of viscosity, ensuring high cell viability, and achieving high resolution. These advantages make it a valuable technique for creating functional tumor models such as exocrine pancreas spheroid models to study cancer initiation.^288^ EBB merges a fluid‐dispensing system with an automated robotic system dedicated to extrusion and bio‐printing.^289^, ^290^ Its capability of creating porous constructs facilitates the engineering of vasculature in tumor models and the manipulation of cancer lymphatics.^291^

#### 3.2.2 3D bio‐printing in tumor immunology investigations

Tumor‐immune cocultures enabled by 3D bio‐printing provide a new avenue for cancer immunotherapy research. 3D bio‐printed tumor models in collagen matrices containing immune cells enable the tracking of immune cell‐tumor interactions and facilitate simulated immunotherapy.^292^ Besides, the ability to accurately measure T cell tumor infiltration demonstrates the potential of 3D bio‐printing as a valuable tool for preclinical characterization and selection of CAR‐T cells. Compared with 2D cocultures, the bio‐printed 3D neuroblastoma model showed high reproducibility and enabled the detection and quantification of CAR‐T cell tumor infiltration.^293^ Additionally, the 3D bio‐printed tumor models provide a more physiologically relevant environment for studying tumor‐immune cell interactions.^294^ For instance, 3D bio‐printing fabricates a miniature brain model by merging glioma cells with macrophages. This model unveiled the ability of glioma cells to attract macrophages and prompt their transitions to TAMs.^294^ 3D bio‐printing also allows for the creation of tumor models that replicate the microenvironment, enabling the study of cell fusion and the development of targeted therapies.^295^, ^296^

#### 3.2.3 Conclusions for 3D bio‐printing

3D bio‐printing methods have shown great potential in generating valuable preclinical models for cancer immunotherapy and allow for the precise placement of tumor and immune cells.^280^, ^281^ Advances in bio‐printing techniques will be crucial in building more physiologically relevant models to study tumor‐immune interactions.^280^

### 3.3 Reconstruction of tumor‐immune interactions in in vitro models

To apply 3D tumor‐immune coculture systems for TME and immunotherapy research, it is crucial to simulate in vivo tumor‐immune cell interactions. Tumor‐immune interactions can be divided into direct interaction relying on cell contacting and remote interaction relying on mediator secretion.

#### 3.3.1 Simple direct interaction

Direct interaction refers to the interactions in which tumor cells and immune cells are in direct close contact, taking more account of the contact‐depending cytotoxic effects rather than remote interaction based on cytokine secretion or lymphocyte migration. TILs, primarily composed of T cells, are the most common mononuclear immune infiltrates observed in most patients.^11^ Activated CTLs directly engage in immune killing by direct contact with tumor cells, thereby influencing tumor prognosis.^16^ NK cells play a significant role in the treatment of hematological malignancies, while their cytotoxic effects on solid tumors remain controversial, possibly due to their weaker tumor‐infiltrating capacity.^297^ Myeloid cells, such as TAMs, are a heterogeneous and plastic cell population within the tumor. TAM supports cancer progression and treatment resistance but can also mediate antitumor effects when responding to drugs that enhance phagocytic and oxidative functions.^298^, ^299^

Direct interaction is divided into simple and complex interactions. Simple interaction involves tumor cells and one type of lymphocyte, while complex interaction involves at least one other immunomodulatory cell. Simulations of different interaction patterns process different characteristics and have different corresponding construction architectures (Table 2). The simple direct interaction involves only tumor cells and a single type of lymphocyte.^256^, ^262^, ^268^ It focuses on a specific immune cell and usually uses the constructs of tumor spheroid, submerged model, or microfluid chip. Coculture system has been widely used in preclinical testing and mechanism research of several types of novel immunotherapies such as ICB,^252^, ^300^ High‐affinity newsagents (HAN),^265^ iRGD,^253^ Bi‐mab Antibody,^266^ CAR‐T,^255^, ^267^, ^268^ cytokine‐induced killer cell (CIK),^249^ and Vδ2T.^251^, ^270^

**TABLE 2:** Comparison between different interaction simulations.

Interaction patterns	Construction	Definition	Advantages	Disadvantages	References
Simple direct interaction	Spheroid	Tumor and immune cells in direct contact. Involving only tumor cells and a single type of lymphocyte	Focus on a specific immune cell type. Easy to construct.	Unable to simulate complex TME or remote cell‐cell interaction	^252^, ^255^, ^266^
	Submerged				^257^, ^300^
	Microfluid chip				^275^
Complex direct interaction	Spheroid	Tumor and immune cells in direct contact. Including at least two cellular components from the TME.	Able to simulate complex TME and preserve original tumor components.	Complex model construction and unable to simulate remote cell‐cell interaction	^243^
	Submerged				^248^, ^260^, ^301^
	Microfluid chip				^28^, ^273^, ^279^
	ALI				^242^, ^244^
Remote interaction	Spheroid	Tumor and immune cells are cultured in different compartment.	Suitable for the simulation of remote interaction and immune cell migration.	Limited direct interaction	^302^
	Submerged				^263^, ^303^, ^304^
	Microfluid chip				^274^, ^277^

Abbreviations: ALI, air–liquid interface model; TME, tumor microenvironment.

A coculture system that focuses on direct interaction is more suitable for generating and screening immune cells with tumor‐specific killing capacity as it does not include immunomodulatory components.^257^, ^264^ Cytotoxic T cells can be generated in a submerged model of colon cancer with dMMR,^257^ and pancreatic cancer,^264^ when T cells from PBMC were added into the coculture system with IL‐2 and IFN‐γ.

#### 3.3.2 Complex direct interaction

Coculture systems focusing on complex direct interaction include at least one other immunomodulatory cell component. The coculture system can be constructed by adding additional immunomodulatory cell components and usually uses the constructs of a submerged model or microfluid chip. Cell components commonly used for complex coculture include various stroma cells and myeloid cells, including endothelium,^273^, ^279^ fibroblasts,^258^, ^305^ myeloid‐derived suppressor cells (MDSCs),^255^, ^260^, ^301^ or macrophages.^260^, ^261^ Different conditional additives can be added into the medium to change the physiological state of cells in the model according to different tumor types and experimental purposes.^306^ Such additives include R‐spondin, Noggin, Wnt3a, and other growth factors crucial for cell growth and differentiation.^241^, ^260^, ^307^ This kind of coculture has been widely used in TME studies involving complex cell interactions, such as cancer–CTL interaction with TAMs^261^ and CAFs.^305^ Stroma components in TME can interact with tumor cells to form fine 3D structures and further influence the infiltration of lymphocytes into tumors.^258^ By coculturing tumor cells, CTL, and MDSC or M2 macrophages, the addition of myeloid components generates an immunosuppressive environment, resulting in a significant decrease in the lethality of T cells.^248^, ^261^

The simulation of complex tumor‐immune interaction can also be established through a holistic approach, retaining the immune and stromal components of the original tumor.^28^, ^241^, ^242^ Important cytokines such as R‐spondin, Noggin, epidermal growth factor, Prostaglandin 2, Gastrin,^241^ and IL‐2,^259^ should be added to the PDO culture system to better preserve native immune components (Table S1). ALI models have advantages in preserving immune components and have been widely used for PDO construction and immunotherapy testing.^242^ In addition to ALI models, spheroid‐based submerged models,^241^ and microfluid chips^28^ can preserve original immune components under suitable culture conditions and operations. Thus, validation of the efficacies of immunotherapy^241^, ^243^, ^259^ and testing of novel immunotherapies^28^, ^244^ can be carried out. Besides, a complex coculture system that preserves the original immune components can be used in the study of the tumor immunity process and mechanism.^28^, ^259^

#### 3.3.3 Remote interaction

Remote interaction refers to the interplay in which tumor cells and immune cells are not in direct contact. For instance, T cell migration in response to chemokines and adhesion molecules plays a critical role in tumor immunity. This migration is facilitated by the activation of specific signaling pathways including chemokine receptor signaling and further contributes to the antitumor activities. B cells as the second population of tumor‐infiltrated immune cells, possess complex functions encompassing antibody production and immune function regulation.^308^ The coculture model separates tumor cells from immune cells physically and requires lymphocytes to migrate to tumor cells or release cytokines or antibodies. Due to the advantages of creating cytokine gradients, the microfluid chip has been widely used in in vitro immune cell migration studies.^277^ A separated submerged model, in which tumor cells and immune cells are separately cultured in different chambers and separated by a cell‐permeable membrane, can also be used for the study of cell migration.^270^, ^302^, ^304^

A coculture system can be used to simulate remote interactions between tumor cells and immune cells.^263^, ^274^, ^303^ For example, the coculture of tumor cells and endothelial cells using a chambered submerged model can be used to study the efficacies of BCG therapy on immune cell proliferation and cytokine secretion.^263^ Besides, the chambered submerged model can be used as an in vitro simulation of B cell immunotherapy.^303^ Through the utilization of organotypic slice cultures of breast cancer tissue and lymph node tissue, it has been observed that lymph node slices cocultured with tumor slices exhibit greater immunosuppression compared with those cocultured with healthy tissue.^274^

#### 3.3.4 Conclusions for tumor‐immune interaction reconstructions in vitro

The reconstruction of tumor‐immune interactions in 3D in vitro models involves both direct interactions and remote interactions. Direct interactions focus on T and NK cells. Coculture systems with different architectures can be employed for therapies that rely on direct cytotoxicity or to generate tumor‐reactive T lymphocytes.^257^, ^264^ Additionally, the inclusion of immunomodulatory cells such as MDSCs, TAMs, and CAFs contribute to the reconstruction of complex direct interactions.^255^, ^260^, ^301^ Remote interactions encompass T cell migration as well as the secretion of cytokines and antibodies by lymphocytes, which play essential roles in tumor immunity.^308^ Organ‐on‐a‐chip or chambered models can be utilized to investigate these aspects of tumor‐immune interactions.^277^

### 3.4 Preservation of tumor immune microenvironment of in vitro models

The rapid development and extended use of 3D tumor‐immune coculture systems raised a question: whether the tumor‐immune coculture system can restore in vivo tumor immunity. Coculture systems need to be verified from two aspects: tumor cells and immune cells (Figure 1B). To date, most of the verifications have focused on the tumor cell, which can be mainly divided into genetic verification (mutation profile by NGS),^242^, ^257^ molecular biological verification (transcriptome profile by RNAseq),^309^ cytological verification (surface protein profile by IHC),^267^ and functional verification (drug sensitivity tests).^310^ Nevertheless, there is still a lack of knowledge about the preservation of immune cells involved in in vitro tumor‐immune cocultures. Verifications on immune components involve two aspects: cell components and cell functions. For immune cell components, different immune cell subgroups with distinct characteristics can be evaluated based on surface markers using IHC and flow cytometry, which allows for assessing proportional changes of each subgroup. Immune cell functions can be evaluated through experiments measuring cytokine secretion, antibody secretion, and cytotoxicity. Alternatively, experimental immunotherapies can be conducted directly in in vitro models for efficacy observation. Table S2 summarizes the validation of the 3D tumor‐immune coculture system in different cancer types.

#### 3.4.1 Validation of immune cell components

Several studies have shown that PDOs can retain a certain amount of native immune components for a period.^241^, ^243^, ^244^ For the ALI model, IHC staining and other methods have confirmed that organoids can effectively retain a variety of nonimmune cell components including fibroblasts and a variety of lymphocyte components including TILs.^242^ The longevity of lymphocytes can be extended to 60 days with the addition of IL‐2.^242^ Further TCR repertories analysis revealed that TILs in PDOs could effectively reproduce TCR information of TILs in the primary tumor, where TCR components of exhausted T cells are best preserved.^242^ Melanoma‐derived tumor spheroids embedded in a microfluid chip can also retain a variety of immune cell components from the primary tumors.^28^ Spheroid‐based submerged model of human CRC retained a variety of cell components found in the original TME.^311^

However, the proportion of immune cell subsets emerges as different between the original tumor and coculture system. The in vitro coculture model of low‐grade ovarian carcinoma containing PBMC was constructed by a magnetic field, and various lymphocyte components including NK, CD4^+^, CD8^+^, and T_reg_ were retained.^311^ However, the proportion of CD8^+^ T cells was significantly decreased in organoids, while the proportion of T_reg_ was significantly increased.^311^ In another submerged PDO model of human high‐grade serous ovarian cancer, scRNAseq showed that the myeloid component was significantly reduced, while the proportion of lymphocytes (including CD4^+^, CD8^+^, and NK) showed an overall upward trend.^259^ In conclusion, a 3D tumor‐immune coculture system can retain the majority of immune cell types of the original tumor, but the proportion of cell types changes. Myeloid components are reported to generally decrease while the lymphoid components increase.^241^, ^259^ However, CD8^+^ T cells, B cells, and NK cells did not show a clear trend of change, and the underlying mechanisms of changes have also not been elucidated either.

#### 3.4.2 Validation of immune cell function

The verification of immune cell function can be carried out from two perspectives: tumor immunity process reproducibility and immunotherapy efficacy. On the one hand, details of tumor immunity in vivo can be reproduced in the in vitro coculture model to explore the changes in cell composition and physiological state after immunotherapy.^28^, ^259^ After anti‐PD‐1 or anti‐CTLA‐4 treatment on human melanoma organoids, the expressions of CCL19 and CXCL13 were significantly up‐regulated, accompanied by significantly increased IFN‐γ, IL‐2, and TNFα secretion.^28^ In 2021, Wan et al.^259^ performed scRNAseq analysis on high‐degree ovarian cancer organoids before and after ICB treatment. ICB induced an increase in the number of certain immune cell populations, such as CD4^+^/CD8^+^ cells with high expression of CD107. Gene expression analysis further revealed increased cytotoxicity in T and NK cells and decreased exhaustion in T cells.^259^

On the other hand, the immune function of the coculture system can be verified by analyzing the efficacies of immunotherapy in the in vitro coculture model.^28^, ^242^, ^243^ Tumor spheroid from NSCLC patients could preserve original tumor immune components with reactivity to immunotherapy.^243^ Spheroids from the different patients had different characteristics in tumor immune and responded differently to the same immunotherapy.^243^ Spheroid‐based microfluid chip can retain original components including B cells, CD4^+^ T cells, CD8^+^ T cells, and myeloid components including MDSCs, DCs, and TAMs. PDOs can effectively reproduce the sensitivity (MC38) or resistance (Lewis lung carcinoma, B16F10) of the tumor to ICB therapy.^28^ However, the stability and prediction accuracy of the in vitro coculture model for the response of a specific individual to certain immunotherapy remains unclear. It is necessary to conduct studies that compare the response of immunotherapy between the original tumors and the corresponding tumor organoids.

#### 3.4.3 Conclusions on tumor immune microenvironment preservations

Several studies have demonstrated that PDOs can preserve various immune components and their functions.^28^, ^241^ However, several key challenges still exist. Further research is still needed to increase the complexity and reproducibility of immune cells in reconstructed TME. Additionally, the preservation of immune cell function in current models is still limited, hindering the study of the long‐term effects of immunotherapy.^242^

### 3.5 Applications of in vitro tumor models in cancer immunology and immunotherapy

**FIGURE 4:**
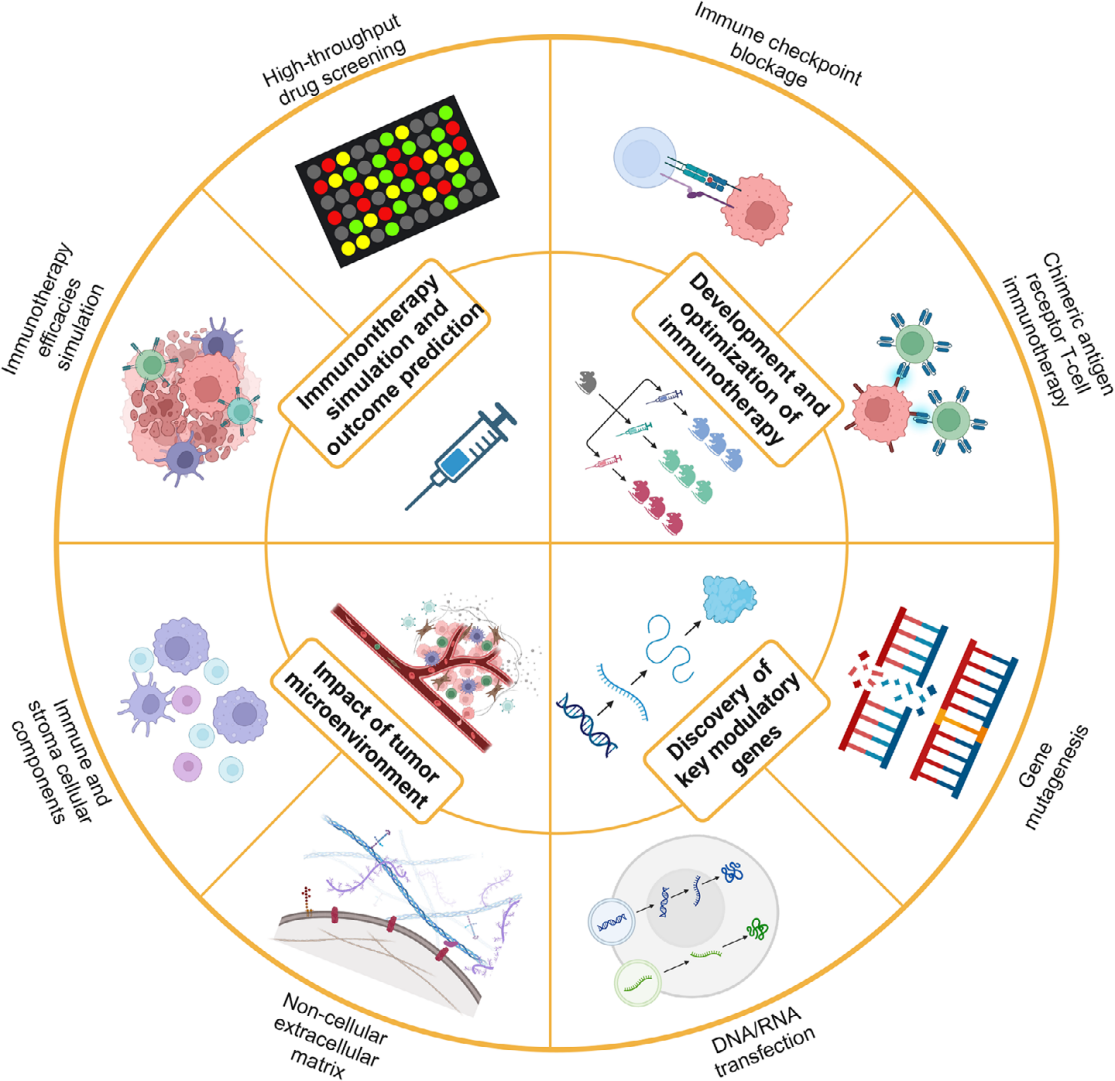
Application of 3D in vitro tumor‐immune coculture system on tumor immunology and tumor immunotherapy. Mechanisms and major influencing factors of tumor immunotherapy and tumor‐immune interactions are the main applications of the 3D in vitro tumor‐immune coculture system, which can be summarized in four aspects in the inner circle: immunotherapy simulation and prediction, immunotherapy optimization, tumor microenvironment factor analysis, and discovery of key modulatory genes. The eight parts of the outer circle are further subdivisions the four aspects of the inner circle. Figure was created with BioRender.com.

Applications of 3D in vitro tumor‐immune coculture systems focus on the mechanisms and key influence factors of tumor immunotherapy and tumor‐immune interactions (Figure 4). By controlling various factors such as immune cell populations, cytokines, and antibodies, models provide a controlled environment to assess the potential outcomes of immunotherapies. Moreover, in vitro models contribute to the development and optimization of immunotherapy by facilitating the screening of potential therapeutic targets and the evaluation of drug candidates. By changing key components of the TME, such as ECM, stromal cells, and immune cells, researchers can investigate the mechanisms underlying immune evasion and therapy resistance. Furthermore, by manipulating gene expression or using gene‐editing techniques, researchers can investigate the functional roles of specific genes in the immune response to cancer.

#### 3.5.1 Immunotherapy simulation and outcome prediction

The efficacy of clinical immunotherapies has fostered an exponential interest in the tumor immune microenvironment, which in turn has engendered a pressing need for robust experimental systems modeling patient‐specific tumor‐immune interactions. The efficacies of different immunotherapies can be modeled in vitro by coculturing exogenous immune cells.^248^, ^260^, ^301^ The in vitro coculture system based on exogenous immune cells makes it possible to develop a high‐throughput screening platform.^256^ However, only adding a single kind of exogenous immune cells may not completely restore the complex interaction between tumor and nontumor components in the real TME.

The in vitro tumor immune cell coculture system based on a holistic approach can be used to simulate the ICB efficacies.^28^, ^242^, ^259^ Compared with a coculture system depending on exogenous immune cells, the holistic coculture system has the following three main advantages. (1) The native model can effectively retain various stroma components, myeloid immune cells, and lymphocytes.^259^ (2) The native model extends the duration for lymphocytes to remain active in the coculture system, enabling the study of medium‐ to long‐term ICB efficacies in vitro.^242^ (3) Holistic coculture system simulates the response characteristics of tumors in vivo to ICB with higher recoverability. When PDOs from NSCLC, RCC, and melanoma were treated with anti‐PD‐1 therapy, the proportion of organoids with TIL activation was similar to that of clinical anti‐PD‐1 therapy.^242^

PDO models have been utilized in personalized prediction for chemotherapy and chemoradiation.^312^, ^313^ Precision immunotherapy using PDOs not only necessitates preservation of tumor characteristics but also presents challenges in retaining patient‐specific TME. Models preserving native TME, such as ALI models and organotypic slice culture, represent significant opportunities.^28^ In a study utilizing melanoma PDOs, the feasibility of PDOs as a personalized immunotherapy screening tool was demonstrated by comparing drug sensitivity from tumors with organoids.^314^ Larger‐scale clinical validation studies, particularly parallel validation studies between patients and PDOs, are still needed.

#### 3.5.2 Development and optimization of immunotherapy

The development of 3D tumor‐immune cell coculture has promoted the discovery of new molecular targets in tumor immunosuppressive environments.^252^, ^300^ In 2021, Sui et al.^300^ determined that the *DKK1* gene promotes the killing effect of CD8^+^ T cells through GSK3β/E2F1/T‐bet axis. In another study in 2021, scRNAseq analysis of the ALI model of high malignant ovarian cancer targeted *BRD1* gene, which plays an important role in T cell and NK cell state transformation.^259^ Other new molecular targets found or verified in 3D tumor immune coculture are summarized in Table S3.

The 3D in vitro culture of tumors preserves the surface antigen characteristics of tumor cells, making it an ideal preclinical testing platform for the application of CAR‐T therapy in solid tumors.^267^, ^268^, ^277^ Combining CAR‐T, CRISPR, and microfluid technology, Preece et al.^277^ tested the killing ability of Hepatitis B‐eTCR^–^/rTCR^+^‐CAR‐T on HepG2 cells in microfluid chips. Knockdown of eTCR upregulated the expression of rTCR and enhanced cell migration and cytotoxic killing effect on tumors.^277^

In vitro 3D tumor‐immune coculture technology is also widely used in the testing of a variety of immunotherapies involving immune cell activation, recognition, and killing. Therapies that activate immune cells through direct stimulation include CIK cell,^249^ BCG vaccine,^263^ nanoparticles,^244^ nanoformulated zoledronic acid,^270^ zoledronate,^251^ and so on. Additionally, by coculturing T cells with antigen‐presenting cells loaded with specific tumor antigens, antigen‐based immunotherapy can be tested.^265^, ^266^ After the bifunctional iRGD‐anti‐CD3 peptide is transmitted into T cells, CTLs targeting specific antigens are generated, and it has a strong killing and penetrating ability to the 3D culture of gastric cancer.^253^ The amphiphilic antibody Bi‐mab enhances the killing ability of lymphocytes to breast cancer PDO by binding tumor cells and lymphocytes respectively.^266^

#### 3.5.3 Impact of TME on cancer immune response

3D tumor‐immune coculture, especially the coculture system involving more than one type of immunomodulatory component, can study the effects of microenvironment factors on tumor immunity.^258^, ^261^, ^262^ The nature of ECM has an impact on tumor immunity and serves as a potential target for tumor immunotherapy. By changing the material and density of the matrix in the in vitro coculture system, tumor immunity in different hardness ECM can be simulated.^262^, ^273^, ^315^ Oxygen concentration is altered in a coculture system for the hypoxic environment simulation.^273^, ^305^ In normal tissue, the oxygen partial pressure (PO_2_) is typically around 65 mmHg, ranging between 12.5 and 96 mmHg. For pathological conditions, cancer tissue usually exhibits lower PO_2_ levels around 10 mmHg and varies between 0 and 95 mmHg.^316^ In comparison, organoids are typically constructed and cultured in CO_2_ cell incubators where PO_2_ is maintained at approximately 150 mmHg, indicating a significant difference in oxygen conditions. A hypoxic environment induces the production of endothelial components in the TME and ultimately leads to the decrease of lymphocyte killing ability, and even lymphocyte apoptosis.^273^ Glucose restriction often occurs before and after eating. After T cell extraction, glucose restriction and re‐supply were carried out in vitro to simulate the change in glucose concentration.^315^ After transient glucose restriction, the immunosuppressive characteristics of T cells decreased while the lethality of tumor organoids increased significantly.^315^

For the cellular components in the TME, current research is mainly concentrated on fibroblasts,^258^ macrophages,^261^ endothelial cells/vascular epithelial cells,^273^, ^279^ and MDSC.^248^, ^301^ Notably, the addition of fibroblasts will change the cell composition and microenvironment architecture of the tumor immune microenvironment.^258^, ^305^ Fibroblasts form a marginal envelope around the coculture system as a microenvironment skeleton, preventing PBMC from infiltrating into the spheroid center.^258^ Endothelial cells can spontaneously form vascular‐like structures in the coculture system, simulating the vascular environment in vitro.^279^ Tumor cells form cell groups with different distributions and sizes according to their subtypes and further affect the migration and infiltration of immune cells.^279^

#### 3.5.4 Discovery and validation of key immune‐modulatory genes

A combination of in vitro tumor immune cell coculture and gene expression manipulation can directly examine the role of genes in tumor immunity.^248^, ^276^, ^301^ Mutagenesis, RNA interference (RNAi), and other technologies specifically inhibit or knock out the expression of specific genes in cells and can be applied to the study of gene function. The glioma‐associated oncogene (*GLI*) related Akt–mTOR pathway directly affects the expression of PD‐L1 in the formation of the immunosuppressive environment of gastric cancer.^248^ The knockout of *HER2* inhibits the expression of the Akt–mTOR pathway and PD‐L1 gene and effectively increases the sensitivity of gastric cancer organoid tumors to anti‐PD‐1 therapy in vitro.^301^ Breast cancer is one of the more common cancers that metastasize to the brain. By constructing a BBB in vitro in microfluidic chips, Mustafa et al.^276^ found that the response of tumor cells to T cells was crucial to the development of brain metastasis, especially with *GBP1* overexpression.

On the other hand, we can increase the expression of specific genes in cells by virus transfection and other methods and observe the effect of increased gene expression on tumor immunity.^304^, ^307^ Meng et al.^304^ used gastric cancer cells transfected with CXCL10 expression and T cells induced with CXCR3 expression for the construction of a tumor‐immune coculture system and directly observed the dose‐dependent effect of T cell migration and infiltration and CXCL10–CXCR3 interaction intensity. Elmira Asl et al.^307^ used a 1:1 ratio of tumor cell‐T cell coculture to induce T_reg_ production in vitro. The miR124 indirectly inhibits the differentiation trend of T_reg_ and weakens the generation of an immunosuppressive environment by mediating the decrease of STAT3 expression on the tumor surface.^307^

#### 3.5.5 Conclusions for in vitro tumor model applications

The use of spheroid and submerged models as immunotherapy testing platforms has been widely adopted due to their efficiency in immunotherapy simulation and outcome prediction.^248^, ^260^, ^301^ Additionally, models preserving stromal and immune components, represented by the ALI model, replicate the native TME and thus simulate the ICB response of in vivo tumors with a higher recoverability.^28^, ^242^, ^259^ Besides, high manipulability of in vitro models allows for the investigation of various TME factors. Organ‐on‐a‐chip has gained attention for its ability to faithfully reproduce complex TME structures.^273^, ^276^, ^279^ Combined with gene editing techniques and RNAi, these models enable the exploration of gene functions within the TME.^248^, ^276^, ^301^

## 4 IN VIVO MOUSE MODEL FOR TUMOR IMMUNOLOGY INVESTIGATION

**FIGURE 5:**
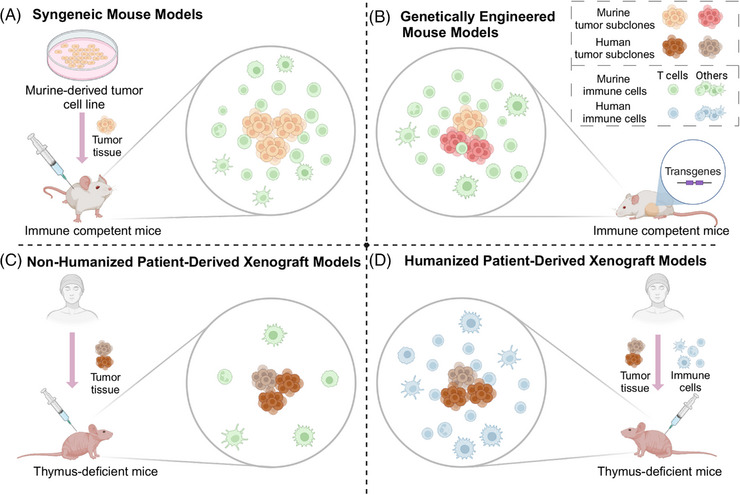
Comprehensive comparison of in vivo models in terms of tumor microenvironment reconstructions. (A) Syngeneic mouse models have the highest usability and the broadest applicability but are limited by factors including low tumor heterogeneity and the absence of tumor evolution processes. (B) Genetically engineered mouse models allow for the monitoring of the entire tumor development process, facilitating the study of gene contributions to tumor formation, but are still limited by the disparities between mice and human patients. (C) Patient‐derived xenografts maintain genomic diversity, tumor structure, and microenvironment of the human tumor. However, evaluating their utility requires careful consideration of host mice immunodeficiency levels. (D) Humanized patient‐derived xenografts facilitate the development of personalized immunotherapy, particularly in simulating the personalized tumor microenvironment. Figure was created with BioRender.com.

With an increasing interest in the advancement of efficient immunotherapies, the creation of immune‐competent mouse models that accurately replicate human diseases appears to be a key challenge.^317^ In the context of developing standard cytotoxic cancer therapies, xenotransplantation models serve as an industry gold standard in which human cancer cell transplantations are entailed into immunocompromised mice to evaluate the effectiveness and safety.^317^ However, the development of immunotherapies further necessitates systems possessing a completely functional immune system characterized by heterogeneity and adaptability, enabling constant adaptation and evolution along with the tumor.^318^ Consequently, a crucial criterion for assessing a preclinical mouse model is its ability to mimic human cancer progression, encompassing the faithful reproduction of cancer genomic heterogeneity, as well as the establishment of an intricate TME housing substantial populations of immune and stromal cells (Figure 5).^318^

### 4.1 Syngeneic tumor model

Syngeneic tumor models are the most widely used choice in in vivo preclinical studies.^318^ By utilizing inbred strains, tumor cell lines are isolated and expanded in vitro and subsequently transplanted to establish tumor‐bearing systems.^318^ One of the key advantages is the high usability. By employing cell lines that can rapidly expand into consistent and large quantities, these models enable studies and databases that require substantial sample sizes, which can be challenging to achieve with genetically engineered models.^319^, ^320^, ^321^ Moreover, syngeneic tumor models offer the opportunity to genetically manipulate the cells, allowing for the evaluation of specific biomarkers associated with immunotherapy sensitivity or resistance.^320^, ^321^, ^322^ Additionally, researchers can assess the impact of various factors on the efficacy of immunotherapeutic approaches.^320^, ^323^, ^324^, ^325^

However, while syngeneic tumor models provide a stable biological nature of tumor grafts, they lack the genomic and microenvironmental heterogeneity that characterizes human tumors, including heterogeneity among different patients and heterogeneity within the same patient.^13^, ^326^ Fundamentally, this is due to the absence of the cancer stem cells, and the complex clonal evolution process from stem cells to the entire tumor.^327^ To address this issue, one possible solution is to inject multiple lineages to generate tumors composed of multiple populations. For instance, when injecting SCLC into mouse models as a mixed population, the mesenchymal cells conferred metastatic capability to the neuroendocrine cells, thereby highlighting the significance of tumor cell heterogeneity in determining tumor properties.^328^ On the other hand, syngeneic tumor models possess higher usability and efficiency compared with other models but lack the natural steps of tumor evolution.^329^ To address this issue, tumor cells can be injected into the corresponding organ locations of mice with specific background diseases.^330^ Additionally, the rapid growth rate of tumors in these models shortens the latency period, providing an inadequate time window to evaluate the efficacy of immunotherapy and making it impossible to study the early stages of cancer development.^331^, ^332^

### 4.2 Genetically engineered mouse model

GEMMs utilize transgenic mice with specific alleles, enabling the natural progression of malignancies in immunocompetent animals.^333^ These models commonly employ tissue‐specific promoters to activate oncogene or induce deletion of tumor suppressor genes using recombinase enzymes.^334^, ^335^ By introducing these genomic alterations, GEMMs replicate the development of invasive cancers as well as precancerous lesions.^336^, ^337^ The extended period of tumor development in these models also provides a longer timeframe for immunotherapeutic interventions, which are important to elicit an effective antitumor immune response.^338^ As GEMMs initiate the neoplastic conversion of healthy cells within the appropriate organ site, the stepwise evolution and advancement of cancer enable the establishment of a multifaceted TME, encompassing both immunosuppressive conditions and stromal vasculature.^333^ Moreover, these models can be designed to mimic alterations in genes that impact the TME and, consequently, influence the immunotherapy efficacy. For example, by employing models in which tumor growth is induced by *Pten* deficiency, scientists can assess treatment strategies that augment the vulnerability of these tumors to immunotherapeutic interventions.^339^

However, GEMMs have several limitations. The occurrence of deleterious mutations impacting multiple target cells at the organism or tissue level can lead to premature mortality in the model.^340^ Furthermore, the tumor mutational burden observed in GEMMs may not precisely mirror that observed in the corresponding human cancer.^341^, ^342^ This is particularly crucial when assessing the efficacy of immunotherapies, as a higher mutational burden is a significant factor in evaluating the effectiveness of immune checkpoint blockade.^343^ On the other hand, the incomplete penetrance of mutations in GEMMs leads to delayed cancer onset, causing nonsynchronous tumor occurrence among mice.^344^ The development of nongermline GEMMs, conditional GEMMs, and advancements in genome editing technologies like CRISPR‐Cas9 have helped overcome these limitations.^29^, ^335^, ^345^ As an illustration, when delivering potent tumor suppressors (*Tp53* and *Cdkn2a*) targeted sgRNAs and Cas in GEMM liver, tumor formations were only observed when additional triggers existed, including *Kras* G12D mutation and CCl_4_ related inflammation.^346^

### 4.3 Patient‐derived xenograft

PDXs are preclinical models created by injecting human tumor cells or implanting tumor tissue into immune‐deficient animal hosts. PDXs preserve the genomic heterogeneity, tumor architecture, and microenvironment factors of the primary tumor, making them valuable for evaluating therapeutic efficacy in vivo.^347^ The success rate of establishing PDXs relies on various factors, including the animal species, cancer type, and specific implantation technique.^348^ Metastatic tumors with aggressive behavior tend to have a higher engraftment success rate. Certain tumor types, such as colorectal or gastric cancer, have a higher success rate compared with others, like breast or kidney cancer.^348^, ^349^, ^350^

Immunodeficiency levels in host mice are another critical consideration when assessing its applicability. Traditional athymic nude mice exhibit deficient thymic development and impaired T‐cell function but retain functional innate immune cells, including neutrophils, DCs, B cells, and NK cells. Thereby, they enable the representation of diverse aspects of the immune response.^351^ To better replicate the original TME, researchers have developed humanized animal models.^352^, ^353^ These models involve the combination of reconstituted human hematopoietic systems with tumor samples. Immunodeficiency mice reconstituted with human CD34^+^ umbilical cord blood cells induce PDX regression when treated with anti‐PD‐1 therapy.^354^ These responses were based on the combinations of human hematopoietic cells and allogeneic PDX, regardless of the degree of human leukocyte antigen (HLA) differentiation between hematopoietic stem cells (HSCs) and PDX.^354^ Another approach to humanize mouse immune system is to use TILs from the same tumor.^355^ Tumor cells and TILs from the same patient were transplanted sequentially into immune‐deficient mice and the demonstrations of antitumor activity were evaluated between responders and nonresponders.^355^ Alternatively, modifying the host mice is another strategy to enhance the expansion of human immune cells.^356^, ^357^ As an exemplification, NSG‐SGM3 mice transgenically expressing human stem cell factors were found to have an improved human B cell development and function after human HSCs engraftment.^356^ However, the impact of these modifications on the antitumor response in reconstituted mice remains unclear and requires further investigation.

### 4.4 Preservation of tumor immune microenvironment in in vivo models

To establish an effective testing platform for tumor immunotherapy in mouse models, it is crucial to reconstruct the TME that resembles the human tumor setting. GEMMs provide a valuable tool for studying tumor development in immunocompetent animals, allowing for the investigation of the TME.^29^ However, the use of GEMMs for testing immunotherapy is limited by the potential cross‐reactivity between murine and human targets, particularly when agents require antigen presentation by human MHC class I.^355^ To address this limitation, GEMMs incorporating human MHC class I and MHC class II have been developed to assess peptide‐specific T‐cell responses relevant to human antitumor immunity.^358^ Additionally, GEMM models have been created to express target antigens, bridging the gap between human and mouse tumor antigens, and enabling a more accurate evaluation of antigen‐specific immunotherapies.^333^ However, differences in antigen‐presenting mechanisms between humans and mice still exist, presenting potential challenges for future applications.

Another approach for reconstructing the TME in mouse models is using humanized models. Humanized models involve the engraftment of human immune cells into immunodeficient mice, allowing for the presence of both human tumor cells and human immune components. Humanized models can be generated by introducing PBMCs.^359^ However, despite the feasibility of using PBMCs in autologous tumor‐bearing PDXs, challenges arise regarding the viability of these cells, the need for sequential blood draws from the patients, and the strong graft‐versus‐host reaction.^360^ To address this, CD34^+^ HSCs or other hematopoietic progenitors could be used instead.^354^ Besides, reconstructing the TME in PDX using autologous TILs is an emerging and promising approach. This strategy allows for the preservation of native immune components, thereby maintaining their complexity and functionality.^355^

In addition, efforts have also been made to incorporate other TME components into mouse models. For example, the inclusion of stromal cells, such as CAFs, endothelial cells, and ECM components, can enhance the fidelity of the TME.^361^ These models allow for the investigation of tumor‐stroma interactions, angiogenesis, and the influence of the ECM on tumor behavior.^362^

### 4.5 Applications of in vivo tumor models in cancer immunology and immunotherapy

In recent years, there have been significant advances in cancer immunology and immunotherapy, which have revolutionized cancer treatment. In vivo mouse models play a pivotal role in evaluating the effectiveness of various immunotherapeutic strategies, including ICB and ACT. These models allow researchers to assess the tumor response to immunotherapy, measure immune cell infiltration, and investigate the mechanisms underlying treatment resistance. Furthermore, cancer immunology and TME have gained significant attention in recent years. In vivo tumor models provide a platform to study the complex interactions between cancer cells, immune cells, and stromal components within the TME. These models enable the exploration of key factors influencing tumor progression, immune evasion, and the development of novel immunomodulatory interventions.

#### 4.5.1 Immunotherapy simulation, prediction, and optimization

Syngeneic mouse models are suitable for high‐throughput studies. The TISMO database encompassed 1518 RNA‐seq samples from 68 syngeneic mouse tumor models across 19 cancer types (832 were from ICB studies), providing great convenience for analyzing ICB response and resistance biomarkers.^319^ Additionally, syngeneic mouse models can be used for biomarker research through genetical manipulations. For instance, PET radiotracers targeting CD4 and CD8 were developed and tested in syngeneic mouse models to find that CD4^+^ or CD8^+^ TILs can serve as anti‐PD‐1 therapy biomarkers.^322^

GEMMs utilize transgenic mice with specific gene alterations, allowing for monitoring the natural progression of tumors. This characteristic provides a longer growth period and enables the study of long‐term effects of immunotherapy.^338^, ^363^ For adverse effects, GEMMs with slower tumor kinetics allow for immune‐related adverse events (irAE) development. Prolonged T_reg_ depletion in Foxp3‐DTR mice served as biomarkers for the antitumor responses and irAE severity in ipilimumab/nivolumab‐treated patients.^363^

PDX models based on humanized mice have rapidly developed and offer unique advantages in simulating personalized TME for immunotherapy investigations.^347^ Moreover, the utilization of patient‐derived TILs allows for the study of ACT in humanized mouse models.^355^ Tumor cells and TILs from the same patient were transplanted sequentially into immune‐deficient mice. TILs derived from ACT responders demonstrate antitumor activity, whereas TILs from nonresponders did not.^355^

#### 4.5.2 Cancer immunology and TME investigation

By utilizing syngeneic mouse models, researchers can explore various factors influencing the process of tumor immunity.^320^ By utilizing three different syngeneic mouse models, distinct TME with different ICB response was constructed, demonstrating that increased TIL levels were related to high ICB sensitivity.^320^ GEMMs have emerged as powerful tools for studying cancer progression, including the development of precursor lesions and the impact of various factors on tumor initiation and growth.^333^, ^337^ As an example, the concurrent expression of *Trp53* R172H and *Kras* G12D in mouse pancreatic tissue resulted in the cooperative formation of invasive and highly metastatic carcinoma of pancreatic ductal adenocarcinoma.^337^

PDX models involve the transplantation of patient tumor tissues into immunodeficient mice, allowing for the growth of tumors that retain the patient's original tumor characteristics and TME components. Therefore, PDX models provide a platform to study the interactions between tumor cells and the immune system in a personalized context.^347^ Notably, CTCs have been employed to create animal models called cell line‐derived tumor xenografts (CDXs), which offer a distinct opportunity to assess the genomic characteristics of metastatic tumors.^364^ CDX models were created by enriching CTCs from four SCLC patients and injecting them into animals. Genomic analysis of CDX models revealed preservation of the original mutational profile, and they also exhibited similar therapeutic responses as observed in patients.^365^ Nevertheless, several limitations exist for this approach including technical challenges in isolating and expanding CTCs, the absence of noncancerous components, and the distinguishment of different metastatic sites.^222^

#### 4.5.3 Conclusions for in vivo tumor model applications

Compared with in vitro tumor models, in vivo mouse models’ advantage is possessing a functional immune system. However, it is important to acknowledge that a single type of model can only replicate an aspect of the in vivo TME. Therefore, developing new preclinical models or using multiple experimental models is necessary to complement each other's strengths and provide a more comprehensive understanding of cancer immunology.^318^

## 5 DISCUSSION

Biomarkers serve as a crucial link between the unobservable immune status and the observable therapy response. In clinical applications, there is a growing demand for biomarkers that enable effective prediction and real‐time reflection of tumor status.^2^ While PD‐L1 and MSI have been recommended by current clinical guidelines,^88^, ^138^ their efficacy remains limited.^97^, ^144^ In recent years, several novel biomarker candidates have emerged, including tumor neoantigens and biomarkers detected by liquid biopsy.^38^, ^69^ Tumor neoantigens, which are directly associated with immune recognition and T cell‐dependent cytotoxicity, have demonstrated effectiveness in various immunotherapies.^37^, ^38^ However, a significant challenge lies in accurately defining high‐quality neoantigens, which can be addressed through the application of emerging AI techniques that predict the relationships between protein structure and immunogenicity.^366^ Biomarkers from liquid biopsy provide a noninvasive means of real‐time monitoring.^69^ However, due to the low signal‐to‐noise ratio, it is crucial to employ noise reduction, decontamination, and feature extraction techniques. This necessitates the use of detection methods and prediction approaches with high sensitivity and specificity.^227^, ^230^

Biomarkers can reflect key aspects of the complex tumor immune processes, making them widely applicable in mechanism studies. For instance, surface markers and TILs can be used to evaluate the in vitro constructed TME, thereby aiding in the creation of a model that accurately reflects the real TME.^242^ Compared with 2D models, 3D models offer a more comprehensive representation of the TME and can currently incorporate various tumor‐immune interactions.^28^, ^241^, ^242^ However, challenges remain, including the need for improved accuracy in TME reconstruction, the alterations in cell type proportions over time, and the preservation of cell functionality.^242^ In terms of animal models, the latest advancement involves reconstructing the human immune system in immune‐deficient mice.^359^ However, due to insufficient TME reconstruction, long cultivation time, and low success rate, the clinical application of animal models is still limited.^318^

Despite their widespread application, current experimental models are still far from fully replicating TME. Emerging technologies present an opportunity for the development of novel experimental models.^244^, ^279^, ^346^ For instance, the ALI models allow direct tumor tissue culture, thereby avoiding cell damage during tissue processing and better preserving the diverse cell types within the model.^242^, ^244^ Organ‐on‐a‐chip utilizes microfluidic technologies to create 3D models that closely mimic the blood flow environment.^279^ Tissue slice culture facilitates the simulation of interactions between two whole organs rather than isolated cells.^274^ 3D bio‐printing implements the precise construction of the TME.^280^ Additionally, the CRISPR‐Cas9 gene editing technology has revolutionized the construction of humanized mouse models, making the process more convenient and efficient.^346^

In conclusion, advancements in cancer immunology hold great potential for the development of efficient, accurate, and real‐time biomarkers, as well as experimental models that faithfully replicate the TME. With biomarkers acting as the bridge and experimental models serving as the platform, we will enter a new era of truly efficient and personalized cancer immunotherapy.

## AUTHOR CONTRIBUTIONS

Hengyi Xu, Ziqi Jia, Jianzhong Su, and Jiaqi Liu contributed to the initial idea and conceptualization of the review article. Hengyi Xu, Ziqi Jia, Fengshuo Liu, and Jiayi Li were responsible for writing the initial draft of the review article. Hengyi Xu, Jiayi Li, Yiwen Jiang, Yufan Yang, and Yansong Huang prepared the figures. Pengming Pu, Pengrui Tang, Tongxuan Shang, Yufan Yang, and Yongxin Zhou prepared the tables. Hengyi Xu, Ziqi Jia, Fengshuo Liu, and Yufan Yang prepared the revision. Jiaqi Liu and Jianzhong Su reviewed the manuscript. All authors listed have made a substantial contribution to the work. All authors have read and approved the article.

## CONFLICT OF INTEREST STATEMENT

The authors declare no conflict of interest.

## ETHICS STATEMENT

Not applicable.

## Supporting information

Supporting information

## Data Availability

Not applicable.
