# Supplementary material for: Biomarkers and experimental models for cancer immunology investigation
Source: MedComm (2020). 2023 Dec 2;4(6):e437. doi: 10.1002/mco2.437 (PMC10693314; doi:10.1002/mco2.437)
Supplement: Supplementary file 1 — Supporting information [file MCO2-4-e437-s001.docx]

**Supplementary Materials for**

**Biomarkers and Experimental Models for Cancer Immunology Investigation**

Henyi Xu^#^, Ziqi Jia^#^, Fengshuo Liu^#^, Jiayi Li^#^, Yansong Huang, Yiwen Jiang, Pengming Pu, Tongxuan Shang, Pengrui Tang, Yongxin Zhou, Yufan Yang, Jianzhong Su^*^, Jiaqi Liu^*^

Correspondence to: j.liu@cicams.ac.cn; sujz@wmu.edu.cn

This file includes:

Table S1

Table S2

Table S3

# Table S1. Cytokines used in 3D tumor-immune coculture system

| **Cytokines** | **Target cell types** | **Mechanism** | **Ref** |
| --- | --- | --- | --- |
| EGF | Cancer; Native immune | Activate MAPK pathways | 1-4 |
| FGF10 | Cancer | Fibroblast growth factor family | 1, 2 |
| FGF2 | Cancer | Fibroblast growth factor family | 1 |
| Gastrin | Cancer; Native immune | Activate JNK to promote c-Jun activity | 1, 2, 4 |
| GM-CSF | PBMCs; MDSC | Activate JAK/STAT pathway | 1, 2, 5 |
| Hydrocortisone | Cancer | Activate MAPK-mediated signaling pathway | 1 |
| IFNgamma | Cancer; Native immune; PBLs; Macrophage; T | Activate JAK/STAT1/IRF1 pathway | 6-9 |
| IL-15 | PBMCs; T; NK | Activate JAK/STAT, PI3K and MAPK pathways | 10-13 |
| IL-1α | PBLs | Activate NK-κB and MAPK pathways | 14 |
| IL-1β | PBMCs; MDSC | Activate NK-κB and MAPK pathways | 1, 2, 5 |
| IL-2 | Native immune; PBMCs; PBLs; Vδ2 T; CD8+ T; T; NK | Activate JAK/STAT, PI3K and MAPK pathways | 4, 6, 15-17 |
| IL-21 | PBMCs; T | Activate JAK/STAT, PI3K and MAPK pathways | 11 |
| IL-4 | PBMCs | Activate JAK/STAT, PI3K and MAPK pathways | 1, 2, 5, 7 |
| IL-6 | PBMCs; MDSC | Activate JAK/STAT, PI3K and MAPK pathways | 1, 2, 5 |
| IL-7 | CD8+ T | Activate JAK/STAT and PI3K pathways | 5, 12, 18 |
| Insulin | Cancer | Activate PI3K and MAPK pathways | 1 |
| Noggin | Cancer; Native immune | Inhibit BMP pathways | 1, 2, 4 |
| PGE2 | Native immune; PBMCs; MDSC; NK | Activate EP1-4 for PLC/AC/PI3K activation | 1, 3, 5, 19 |
| R-Spondin | Cancer; Native immune | Activate the WNT/β‐catenin pathway | 1-4 |
| TGF-β1 | MDSC | Activate SAMD to alter cell transcription | 1, 2, 5 |
| TNF-α | PBMCs; MDSC | Activate JAK/STAT1 and MAPK pathways | 1, 5, 14 |
| VEGF | MDSC; HUVECs | Protein tyrosine kinase activity of receptors | 1, 5, 13 |
| WNT | Cancer; Native immune | Activates classical WNT pathway | 1, 2, 4 |

**EGF** Epidermal growth factor; **FGF** Fibroblast growth factor; **GM-CSF** Granulocyte-macrophage colony-stimulating factor; **IFNgamma** Interferon-gamma; **IL** interleukin; **PGE** Prostaglandin; **TGF** Transforming growth factor; **TNF** Tumor necrosis factor; **PBMC** Peripheral blood mononuclear cell; **DC** Dendritic cell; **MDSC** Myeloid-derived suppressor cell; **NK** Natural killer cell; **HUVEC** Human umbilical vein endothelial cell; **MAPK** Mitogen-activated protein kinase 1; **JNK** c-Jun kinase; **c-Jun** Jun proto-oncogene; **JAK** Janus kinase; **STAT** Signal transducer and activator of transcription; **IRF** Interferon regulatory factor; **PI3K** Phosphoinositide 3-kinase; **NF-κB** Nuclear factor-κB; **BMP** Bone morphogenetic protein; **EP** E-prostanoid; **PLC** Phospholipase C; **AC** Adenylate cyclase; **SAMD** Mothers against decapentaplegic homolog.

# Table S2. Validation of tumor-immune 3D in vitro coculture system of different tumor types

|  | **Tumor genomic validation** | **Tumor transcriptomic validation** | **Tumor translatomic validation** | **Immune cell retention validation** | **Immune cell proportion validation** | **Immune cell function validation** |
| --- | --- | --- | --- | --- | --- | --- |
| Brain | √ | √ | √ | √ | √ | √ |
| Head and neck | √ | √ | √ |  |  | √ |
| Thyroid | √ |  | √ |  |  |  |
| Lung | √ | √ | √ | √ | √ | √ |
| Breast | √ | √ | √ | √ | √ | √ |
| Bone/soft tissue | √ | √ | √ | √ | √ | √ |
| Skin | √ | √ | √ | √ | √ | √ |
| Oral/Esophagus | √ | √ | √ |  |  |  |
| Stomach | √ | √ | √ |  |  | √ |
| Liver/gall bladder | √ | √ | √ |  |  | √ |
| Pancreas | √ | √ | √ | √ | √ | √ |
| Kidney | √ | √ | √ |  |  |  |
| Colorectum | √ | √ | √ | √ | √ | √ |
| Ovary | √ | √ | √ | √ | √ | √ |
| Bladder | √ | √ | √ |  |  | √ |
| Prostate | √ | √ | √ |  |  |  |

# Table S3. Immunotherapy studies based on 3D tumor-immune coculture system

| **Year** | **First Author** | **Interaction patterns** | **MoA** | **Target** | | | **Drug** | | **Ref** | |  |
| --- | --- | --- | --- | --- | --- | --- | --- | --- | --- | --- | --- |
| 2020 | Maas | Simple close | ICB | DNAM-1 | | | Anti-DNAM1 clone 11A8, Biolegend, #338302 | | 13 | |  |
|  |  |  |  | TIGIT | | | Anti-TIGIT hIgG1.3 Fc blocking antibody | | | |  |
|  |  |  |  | CD96 | | | Anti-CD96 clone NK92.39, Biolegend | | | |  |
| 2021 | Gopal | Simple close | ICB | HER2 | | | Trastuzumab | | 20 | |  |
|  |  |  |  | PD-L1 | | | Atezolizumab | | | |  |
| 2021 | Sui | Simple close | ICB | PD-1 | | | Pembrolizumab (Keytruda, Merck & Co.) | | 21 | |  |
|  |  |  |  | DKK1 | | | DKK1 neutralizing antibody (Sinobio) | |  | |  |
| 2020 | Holokai | Complex close | ICB | PD-1 | | | Nivolumab (Selleckchem, A2002) | | 1 | |  |
|  |  |  |  | EpCAM | | | EpCAM antibody (Thermo Fisher Scientific) | | | |  |
| 2018 | Neal | Complex close | ICB | PD-1 | | | Nivolumab (Bristol-Myers Squibb)  Anti-mouse-CD279 (a-PD-1, 29F.1.A12) | | 4 | |  |
|  |  |  |  | PD-L1 | | | Anti-mouse-CD274 (a-PD-L1, B7-H1) | |  | |  |
| 2021 | Hong | Complex close | ICB | PD-1 | | | Pembrolizumab  Nivolumab | | 3 | |  |
|  |  |  |  | PD-L1 | | | Atezolizumab | | | |  |
| 2021 | Wan | Complex close | ICB | PD-1 | | | Pembrolizumab | | 17 | |  |
|  |  |  |  | PD-L1 | | | LY3300054 (Eli Lilly and Company) | | | |  |
| 2021 | Koh | Complex close | ICB | PD-1 | | | Nivolumab (Selleck Chemicals, Houston, TX) | | 5 | |  |
|  |  |  |  | Multiple targets | | | Cabozantinib (Sigma-Aldrich) | | | |  |
| 2021 | Kamer | Complex close | ICB | PD-L1 | | | Durvalumab (AstraZeneca) | | 22 | |  |
|  |  |  |  | CTLA-4 | | | Ipilimumab (Bristol Myers Squibb) | | | |  |
| 2018 | Jenkins | Complex close | ICB | PD-1 | | | Pembrolizumab | | 23 | |  |
|  |  |  |  | CCL-2  CTLA-4 | | | Clone 123616, R&D Systems  Ipilimumab | | | |  |
|  |  |  |  | TBK1/IKKε | | | Compound 1 synthesized by authors | |  | |  |
| 2021 | Yin | Complex close | TIL activation | / | | |  | | 24 | |  |
| 2018 | Varesano | Simple close | Vδ2T; antibody | / | | | Zoledronate, cetuximab | | 25 | |  |
| 2019 | Mascolo | Remote | Vδ2T | / | | | Zoledronate | | 26 | |  |
| 2021 | Zhou | Simple close | Short peptide; ICB | PD-1 | | |  | | 9 | |  |
| 2018 | Dijkstra | Simple close | ACT; ICB | PD-1 | | | Anti-PD-1-blocking antibody (by Merus, Utrecht) | | 6 | |  |
| 2021 | Liu | Simple close | ACT | Tumor  neoantigens | | | |  | 7 | |  |
| 2021 | Meng | Simple close | ACT; ICB | PD-1 | | | Anti-PD-1 (Selleckchem, A2002) | | 11 | |  |
|  |  |  |  | PD-L1 | | | Anti-PD-L1 (Biolegend, 329716) | |  | |  |
|  |  |  |  | TIM3 | | | Anti-TIM3 (Biolegend, 345038) | |  | |  |
|  |  |  |  | TIGIT | | | Soluble protein (Prospec, PRO-2498) | |  | |  |
|  |  |  |  | LAG3  NKG2A | | | Soluble protein (R&D, 2319-L3-050)  Soluble protein (Prospec, PRO-2440) | |  | |  |
| 2017 | Sánchez-Rodríguez | Remote | BCG | / | | |  | | 27 | |  |
| 2021 | Warwas | Simple close | BsAb | TAA | | |  | | 28 | |  |
|  |  |  |  | aCD3 | | |  | |  | |  |
| 2019 | Gonzalez-Exposito | Simple close | BsAb | CEA | | |  | | 29 | |  |
| 2021 | Luangwattananun | Simple close | CAR-T | FRα | | |  | | 12 | |  |
| 2020 | Preece | Remote | CAR-T | HBsAg | | | | | 30 | |  |
| 2021 | Zou | Simple close | CAR-T | HBsAg | | | | | 16 | |  |
| 2021 | Supimon | Simple close | CAR-T | MUC1 | |  | | | | 18 | |
| 2020 | Wang | Simple close | CIK | / | |  | | | | 14 | |
| 2021 | Zhou | Complex close | Epigenetic inhibitors | / |  | | | | | 15 | |

**MoA** Mechanism of action; **ICB** Immune checkpoint blockade; **ACT** Adoptive cell therapy; **BCG** Bacille Calmette-Guerin vaccine; **BsAb** Bispecific monoclonal antibody; **HBsAg** Hepatitis B surface antigen **CAR-T** Chimeric antigen receptor T cell immunotherapy; **CIK** Cytokine-induced killer cell; **DNAM-1** Platelet and T cell activation antigen 1; **TIGIT** T cell immunoreceptor with Ig and ITIM domains; **CD96** T cell-activated increased late expression protein; **HER2** Erb-B2 receptor tyrosine kinase 2; **PD-1** Programmed cell death protein 1; **PD-L1** Programmed death-ligand 1; **DKK1** Dickkopf WNT signaling pathway inhibitor 1; **EpCAM** Epithelial cellular adhesion molecule; **CTLA-4** Cytotoxic T-lymphocyte associated protein 4; **CCL-2** Monocyte chemoattractant protein-1; **TIL** tumor infiltrating lymphocyte; **TBK1** TANK binding kinase 1; **IKKε** Inhibitor of nuclear factor kappa-B kinase ε; **TIM3** T cell immunoglobulin and mucin domain-containing protein 3; **LAG3** Lymphocyte activation gene 3; **TAA** tumor-associated antigens; **CEA** Carcinoembryonic antigen; **CEA-TCB**: Carcino Embryonic Antigen-T Cell Bispecific; **FRα** Folate receptor α; **HERV** Endogenous retroviruses; **MUC1** Mucin

**Reference**

**1.** Holokai L, Chakrabarti J, Lundy J, et al. Murine- and Human-Derived Autologous Organoid/Immune Cell Co-Cultures as Pre-Clinical Models of Pancreatic Ductal Adenocarcinoma. *Cancers (Basel)*. Dec 17 2020;12(12):3816.

**2.** Roshani Asl E, Rasmi Y and Baradaran B. MicroRNA-124-3p suppresses PD-L1 expression and inhibits tumorigenesis of colorectal cancer cells via modulating STAT3 signaling. *J Cell Physiol*. Oct 2021;236(10):7071-7087.

**3.** Hong HK, Yun NH, Jeong YL, et al. Establishment of patient-derived organotypic tumor spheroid models for tumor microenvironment modeling. *Cancer Med*. Aug 2021;10(16):5589-5598.

**4.** Neal JT, Li X, Zhu J, et al. Organoid Modeling of the Tumor Immune Microenvironment. *Cell*. 2018;175(7):1972-1988.

**5.** Koh V, Chakrabarti J, Torvund M, et al. Hedgehog transcriptional effector GLI mediates mTOR-Induced PD-L1 expression in gastric cancer organoids. *Cancer Lett*. Oct 10 2021;518:59-71.

**6.** Dijkstra KK, Cattaneo CM, Weeber F, et al. Generation of Tumor-Reactive T Cells by Co-culture of Peripheral Blood Lymphocytes and Tumor Organoids. *Cell*. Sep 6 2018;174(6):1586-1598.

**7.** Liu T, Tan J, Wu M, et al. High-affinity neoantigens correlate with better prognosis and trigger potent antihepatocellular carcinoma (HCC) activity by activating CD39(+)CD8(+) T cells. *Gut*. Oct 2021;70(10):1965-1977.

**8.** Chandrakesan P, Panneerselvam J, May R, et al. DCLK1-Isoform2 Alternative Splice Variant Promotes Pancreatic Tumor Immunosuppressive M2-Macrophage Polarization. *Mol Cancer Ther*. Jul 2020;19(7):1539-1549.

**9.** Zhou S, Meng F, Du S, et al. Bifunctional iRGD-anti-CD3 enhances antitumor potency of T cells by facilitating tumor infiltration and T-cell activation. *J Immunother Cancer*. May 2021;9(5):e001925.

**10.** Song J, Choi H, Koh SK, et al. High-Throughput 3D In Vitro Tumor Vasculature Model for Real-Time Monitoring of Immune Cell Infiltration and Cytotoxicity. *Front Immunol*. 2021;12:733317.

**11.** Meng Q, Xie S, Gray GK, et al. Empirical identification and validation of tumor-targeting T cell receptors from circulation using autologous pancreatic tumor organoids. *J Immunother Cancer*. Nov 2021;9(11):e003213.

**12.** Luangwattananun P, Junking M, Sujjitjoon J, et al. Fourth-generation chimeric antigen receptor T cells targeting folate receptor alpha antigen expressed on breast cancer cells for adoptive T cell therapy. *Breast Cancer Res Treat*. Feb 2021;186(1):25-36.

**13.** Maas RJ, Hoogstad-van Evert JS, Van der Meer JM, et al. TIGIT blockade enhances functionality of peritoneal NK cells with altered expression of DNAM-1/TIGIT/CD96 checkpoint molecules in ovarian cancer. *Oncoimmunology*. Nov 8 2020;9(1):1843247.

**14.** Wang Z, Li Y, Wang Y, et al. Targeting prostate cancer stem-like cells by an immunotherapeutic platform based on immunogenic peptide-sensitized dendritic cells-cytokine-induced killer cells. *Stem Cell Res Ther*. Mar 17 2020;11(1):123.

**15.** Zhou Z, Van der Jeught K, Fang Y, et al. An organoid-based screen for epigenetic inhibitors that stimulate antigen presentation and potentiate T-cell-mediated cytotoxicity. *Nat Biomed Eng*. 2021;5(11):1320-1335.

**16.** Zou F, Tan J, Liu T, et al. The CD39(+) HBV surface protein-targeted CAR-T and personalized tumor-reactive CD8(+) T cells exhibit potent anti-HCC activity. *Mol Ther*. May 5 2021;29(5):1794-1807.

**17.** Wan C, Keany MP, Dong H, et al. Enhanced Efficacy of Simultaneous PD-1 and PD-L1 Immune Checkpoint Blockade in High-Grade Serous Ovarian Cancer. *Cancer Res*. Jan 1 2021;81(1):158-173.

**18.** Supimon K, Sangsuwannukul T, Sujjitjoon J, et al. Anti-mucin 1 chimeric antigen receptor T cells for adoptive T cell therapy of cholangiocarcinoma. *Sci Rep*. Mar 18 2021;11(1):6276.

**19.** Sherman H, Gitschier HJ and Rossi AE. A Novel Three-Dimensional Immune Oncology Model for High-Throughput Testing of Tumoricidal Activity. *Front Immunol*. 2018;9:857.

**20.** Gopal S, Kwon SJ, Ku B, Lee DW, Kim J and Dordick JS. 3D tumor spheroid microarray for high-throughput, high-content natural killer cell-mediated cytotoxicity. *Commun Biol*. Jul 21 2021;4(1):893.

**21.** Sui Q, Liu D, Jiang W, et al. Dickkopf 1 impairs the tumor response to PD-1 blockade by inactivating CD8+ T cells in deficient mismatch repair colorectal cancer. *J Immunother Cancer*. Mar 2021;9(3):e001498.

**22.** Kamer I, Bab-Dinitz E, Zadok O, et al. Immunotherapy response modeling by ex-vivo organ culture for lung cancer. *Cancer Immunol Immunother*. Aug 2021;70(8):2223-2234.

**23.** Jenkins RW, Aref AR, Lizotte PH, et al. Ex Vivo Profiling of PD-1 Blockade Using Organotypic Tumor Spheroids. *Cancer Discov*. Feb 2018;8(2):196-215.

**24.** Yin Q, Yu W, Grzeskowiak CL, et al. Nanoparticle-enabled innate immune stimulation activates endogenous tumor-infiltrating T cells with broad antigen specificities. *Proc Natl Acad Sci*. May 25 2021;118(21):e2016168118.

**25.** Varesano S, Zocchi MR and Poggi A. Zoledronate Triggers Vδ2 T Cells to Destroy and Kill Spheroids of Colon Carcinoma: Quantitative Image Analysis of Three-Dimensional Cultures. *Front Immunol*. 2018;9:998.

**26.** Di Mascolo D, Varesano S, Benelli R, et al. Nanoformulated Zoledronic Acid Boosts the Vδ2 T Cell Immunotherapeutic Potential in Colorectal Cancer. *Cancers (Basel)*. Dec 31 2019;12(1):104.

**27.** Sánchez-Rodríguez C, Cruces KP, Riestra Ayora J, Martín-Sanz E and Sanz-Fernández R. BCG immune activation reduces growth and angiogenesis in an in vitro model of head and neck squamous cell carcinoma. *Vaccine*. Nov 7 2017;35(47):6395-6403.

**28.** Warwas KM, Meyer M, Gonçalves M, et al. Co-Stimulatory Bispecific Antibodies Induce Enhanced T Cell Activation and Tumor Cell Killing in Breast Cancer Models. *Front Immunol*. 2021;12:719116.

**29.** Gonzalez-Exposito R, Semiannikova M, Griffiths B, et al. CEA expression heterogeneity and plasticity confer resistance to the CEA-targeting bispecific immunotherapy antibody cibisatamab (CEA-TCB) in patient-derived colorectal cancer organoids. *J Immunother Cancer*. Apr 15 2019;7(1):101.

**30.** Preece R, Pavesi A, Gkazi SA, et al. CRISPR-Mediated Base Conversion Allows Discriminatory Depletion of Endogenous T Cell Receptors for Enhanced Synthetic Immunity. *Mol Ther Methods Clin Dev*. Dec 11 2020;19:149-161.
